# Direct Assembly of Micrometer‐Long Polymeric Cylinders in Water via Supramolecular Sticker Engineering

**DOI:** 10.1002/marc.202500478

**Published:** 2025-08-14

**Authors:** Sébastien Berruée, Jean‐Michel Guigner, Cécile Huin, Jan Patrick Calupitan, Laurent Bouteiller, Lydia Sosa Vargas, Jutta Rieger

**Affiliations:** ^1^ Institut Parisien de Chimie Moléculaire (IPCM) Sorbonne Université CNRS Paris France; ^2^ Institut De Minéralogie, de Physique des Matériaux et de Cosmochimie (IMPMC) Sorbonne Université CNRS Paris France; ^3^ Université Evry Paris‐Saclay Evry France

**Keywords:** anisotropic nanostructures, perylene diimide (PDI), RAFT polymerization, self‐assembly, supramolecular structure‐directing unit (SSDU)

## Abstract

We report a direct, solvent‐free method to produce micrometer‐length, well‐organized polymer nanocylinders in water. To achieve this, a hydrophilic poly(*N,N*‐dimethylacrylamide) (PMDAc) was functionalized at one chain‐end with a perylene diimide (PDI) sticker using RAFT polymerization. Two PDI RAFT agents were prepared and studied: one featuring two tri(ethylene glycol) (TEG) units at the PDI bay‐positions and one without. The corresponding PDI‐PDMAc conjugates spontaneously self‐assembled in water, driven by *π–π* interactions made of H‐aggregates, and showed a morphological evolution from cylinders to spheres when increasing the polymer chain length. The introduction of TEG units was found to be important to avoid the clustering of nanocylinders or the formation of ill‐defined assemblies, which were observed in the TEG‐free system. Moreover, we found that the PDI‐TEG_2_‐PDMAc with degrees of polymerization (*DP*_n_) below 24 self‐assembled into micrometer‐long nanocylinders. By heating the aqueous polymer solution, this process can be accelerated and is accompanied by a large increase in viscosity. Fluorescence spectroscopy revealed an excimer emission signal for the PDI polymers in water, with a higher emission for cylinders, suggesting better organization within the PDI H‐aggregates. This strategy provides a sustainable approach for developing functional nanomaterials with precise morphological control, eliminating organic solvents and complex processing.

## Graphical abstract

Engineering order at the nanoscale: Tailored design of PDI and TEG‐functionalized poly(*N,N*‐dimethylacrylamide) enables direct assembly of micrometer‐long nanocylinders in water. TEG prevents clustering, while the polymer chain length governs the morphology. Heating or aging the aqueous solutions increases the length of the nanocylinders formed, increasing the viscosity. This scalable approach offers precise control for sustainable nanomaterials without the use of organic solvents.

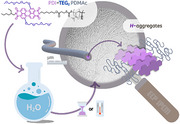

## 1 Introduction

In recent years, π‐conjugated organic materials have regained momentum as promising candidates for optoelectronic applications, thanks to their tunability, lower processing costs, sustainability, and the ability to produce flexible devices; advantages that set them apart from their inorganic counterparts [1, 2]. To tune and optimize their properties, precise control over molecular conformation and long‐range organization is essential [3, 4]. A deep understanding of supramolecular *π–π* interactions is therefore key to achieving long, well‐organized 1D nanostructures [5, 6]. For example, Ghosh et al. used a naphthalene diimide (NDI) supramolecular structure‐directing unit (SSDU), assisted by an amide capable of hydrogen bonding, to drive the self‐assembly of hydrophilic polymers in water towards the cylindrical morphology [7, 8, 9]. Bis‐urea was also used to strengthen NDI aggregation and form polymeric cylinders in water [10]. Other SSDUs, such as squaramides [11], ureido‐pyrimidinone [12], benzene tris‐urea [13], and benzene tris‐peptide [14], were also functionalized at the end/core of the polymer chain to drive their self‐assembly in water towards 1D nanostructures. However, developing long‐range, π‐conjugated 1D nanostructures using eco‐friendly methods, including the use of water as a solvent, is still a current challenge [15, 16].

Among π‐conjugated materials, perylene diimide (PDI) dyes stand out for their exceptional properties, including high thermal and photostability [17]. PDI molecules are well‐known electron acceptors and have already been widely incorporated in optoelectronic devices [18, 19]. Due to their strong *π–π* interactions, their self‐assembling properties in both solution and solid state have been explored [20].

1D PDI assemblies in particular have been obtained in organic solvents, through diverse methods such as heating/aging process [21], sonication [22] or through the use of co‐solvents [23, 24]. Other reported approaches for achieving 1D PDI assemblies include seeding processes in methylcyclohexane (MCH) [25], or in isopropanol:chloroform mixtures (9:1 *i*PrOH:CHCl_3_) [26], phase‐transfer crystallization between hexane and chloroform [27, 28, 29], and interfacial assembly between chloroform and methanol [30, 31]. Additionally, 1D assemblies have been formed by co‐assembling PDI with porphyrins via ionic self‐assembly [32] as well as with pyrene to form organogels in CHCl_3_ [33].

To achieve assemblies in aqueous solutions, co‐solvent methods have been developed. Using such approaches, the formation of helical cylinders from PDI molecules functionalized with carbohydrate groups [34, 35, 36], or micrometer‐long nanocylinders from a (methoxy)bay‐functionalized PDI was reported [37]. Li et al. also demonstrated the formation of 1D, rod‐like structures of micrometer lengths using a PDI dimethacrylate monomer, triggered by slow addition of water to a THF solution and followed by radical polymerization initiated by AIBN [38]. Peptide‐driven assembly of PDIs has also emerged as a promising technique to obtain cylindrical structures in water [39], sometimes facilitated by co‐solvents [40] or ultrasonication [41, 42]. Functionalizing PDI (or other chromophores) with ionic groups [43], neutral hydrophilic groups [44], or polymer chains [45] is an effective strategy to enhance water solubility and enable the formation of such nanostructures in aqueous media. In particular, functionalizing PDI with flexible and water‐soluble poly(ethylene glycol) (PEG) chains or oligo(ethylene oxide), such as triethylene oxide chains, seems promising for steering PDI assembly in water [46]. Rybtchinski et al. developed a series of PEG‐functionalized PDI derivatives that self‐assemble into 1D nanostructures in water, mainly using THF as co‐solvent [47, 48, 49, 50, 51, 52]. In subsequent studies, they also demonstrated that these PEG‐functionalized PDIs can form stable filamentous nanostructures at 10^−4^ mol/L in water [53]. In our recently reported work, we have shown that PDI‐polymer conjugates can be obtained via RAFT polymerization, and spontaneously form ribbon‐like assemblies by simple dissolution in water [54]. Achieving micrometer‐long nanocylinders through direct dissolution in water necessitates the design of an SSDU that strikes a balance: hydrophilic substituents are needed for dissolution in water, but excessively long or bulky substituents can hinder the assembly process [55].

**FIGURE 1:**
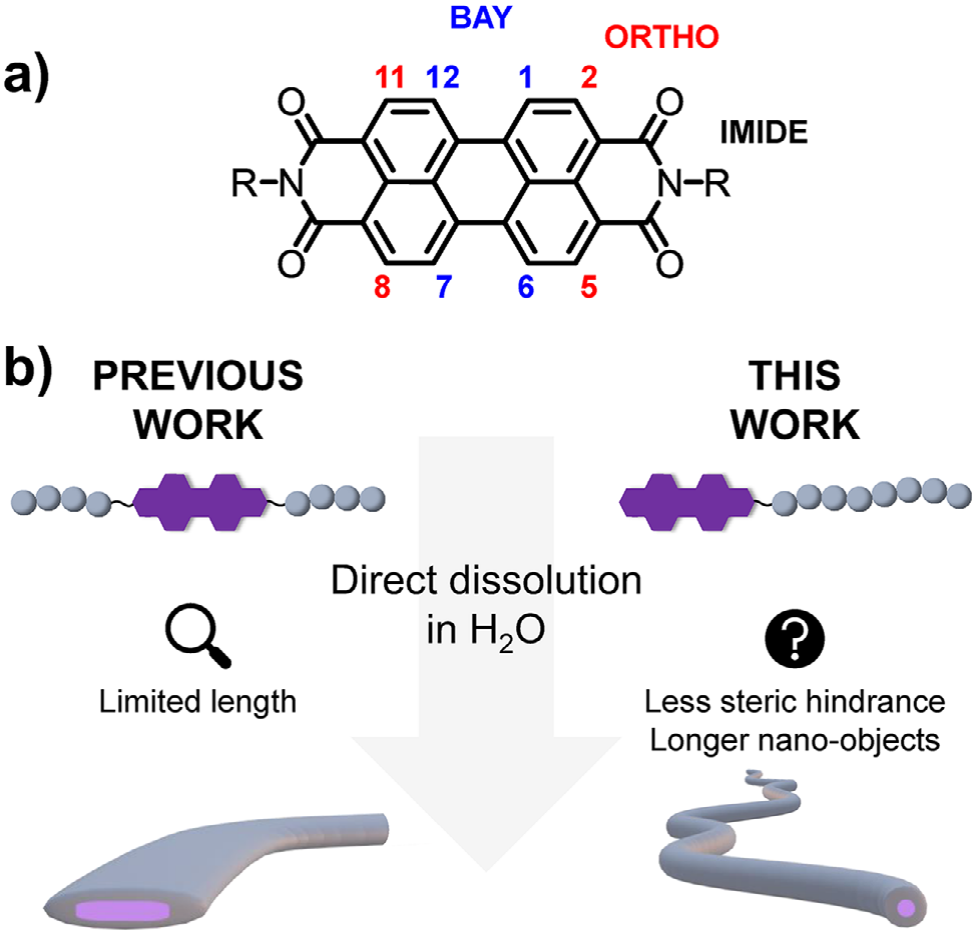
(a) Chemical structure of perylene diimide with the different positions that can be functionalized on the perylene core. (b) Comparison of previous work on PDI inserted in the core of the polymer [54] and this work, where PDI is located at one chain end.

In this work, we aim to design a PDI sticker that serves as an SSDU to drive the formation of micrometer‐long cylindrical (1D) polymeric assemblies by direct dissolution in water. To achieve this, a non‐symmetrical PDI is functionalized on one *N*‐imide position by a universal RAFT agent (Figure 1a), then used in the polymerization of *N,N*‐dimethylacrylamide (DMAc), leading to hydrophilic polymeric chains functionalized on one chain end with the PDI sticker. In contrast to our previously reported systems, where the polymers were grown from both *N‐*positions [54], having the polymer chain attached to only one side of the PDI should reduce the steric hindrance generated by the polymer chain and allow us to form 1D assembly over a greater length (Figure 1b). We studied the morphology and properties of the nanostructures formed in water as a function of the polymer chain length by cryo‐TEM, SAXS, UV/Vis absorbance, and fluorescence emission spectroscopy.

## 2 Results and Discussion

### 2.1 Synthesis and Self‐Assembly of PDI End‐Capped Poly(*N,N*‐Dimethylacrylamide), PDI‐PDMAc

First, a perylene diimide monofunctional RAFT agent, named **PDI‐TTC**, was synthesized in six steps as detailed in Scheme S1. The PDI is non‐symmetrical and bears two different groups at the *N*‐imide positions: an ethylhexyl chain on one side and a trithiocarbonate (TTC) function on the other. The bay positions remain unsubstituted to preserve the planar aromatic structure of the PDI, which is expected to induce its strong aggregation in water [56]. All the procedures and characterizations are reported in the Supporting Information section (Figures S1–S5 and S12a–c).

**SCHEME 1:**
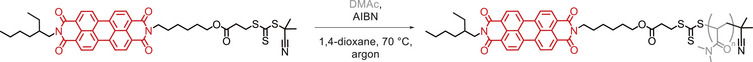
Synthesis of PDI end‐capped PDMAc by RAFT‐mediated polymerization in the presence of PDI‐TTC.

This PDI‐functionalized RAFT agent was then used for the synthesis of a series of PDI‐PDMAcs (Scheme 1, procedure in the Supporting Information). Three polymers with various *DP*_n_ were prepared using the **PDI‐TTC** (Table 1 and Table S1). The polymerizations were deliberately stopped at relatively low conversions to guarantee a very high chain end fidelity. The final polymers were characterized by size exclusion chromatography (SEC) (Figure S14a–c) and ^1^H NMR (Figure S15a–c). Comparing the SEC traces of the RI and UV/Vis detector (at 520 nm, where the PDI strongly absorbs) revealed the incorporation of the PDI into the polymer chain.

**TABLE 1:** Characteristics of the PDI end‐capped PDMAcs: Chemical characterization and results for the self‐assembly in water.

Entry	*DP*_n, NMR_ ^a^	*M*_n, NMR_ ^a^ (kg/mol)	*M*_n, SEC_ ^b^ (kg/mol)	*Đ* ^b^	Morpho. Cryo‐TEM^c^	D_cryo‐TEM_ ^c^ (nm)	Morpho. SAXS^d^	D_SAXS_ ^d^ (nm)	L_SAXS_ ^d^ (nm)
P1	17	2.5	1.9	1.20	Cylinders	7.1 ± 1.6	Cylinders	7.4 (0.21)	*n.d*.
P2	24	3.2	2.6	1.22	Spheres (+Cylinders)	*n.d*.	Spheres (S, 62%) + Cylinders (C, 38%)	S = 12.5 (0) C = 8.6 (0)	C = 45 (0)
P3	134	14.1	8.8	1.42	Spheres	*n.d*.	Spheres	15.4 (0.10)	*n.d*.

^a^ Number‐average degree of polymerization, *DP*_n_, and number average molar mass, *M*_n_, of the purified polymers determined by ^1^H NMR in CDCl_3_.

^b^ *M*_n_ and dispersity, *Đ*, of the purified polymers determined by SEC in DMF (+ LiBr 1 g/L) using PMMA standards.

^c^ Morphology of the nano‐objects identified on cryo‐TEM pictures (Figure 2a–c), with the diameters of the nano‐objects and standard deviations determined using imageJ from cryo‐TEM pictures, with at least 30 measurements.

^d^ SasView models used to fit the SAXS experimental data (Figure 2d–f), with the diameter (D) and length (L) of the nano‐objects determined by fitting data, with lognormal polydispersity in parentheses.

^*^ (0) means that no polydispersity was considered in the fitting.

The three polymers were dissolved in water at 10 g/L. **P2** and **P3** dissolved spontaneously in less than 5 min, and the solutions were stable over time. **P1**, the compound with the lowest *DP*_n_ (17), did not dissolve immediately, but visually dissolved after stirring overnight (at 20°C), while the formation of a precipitate was observed over time in the absence of stirring.

**FIGURE 2:**
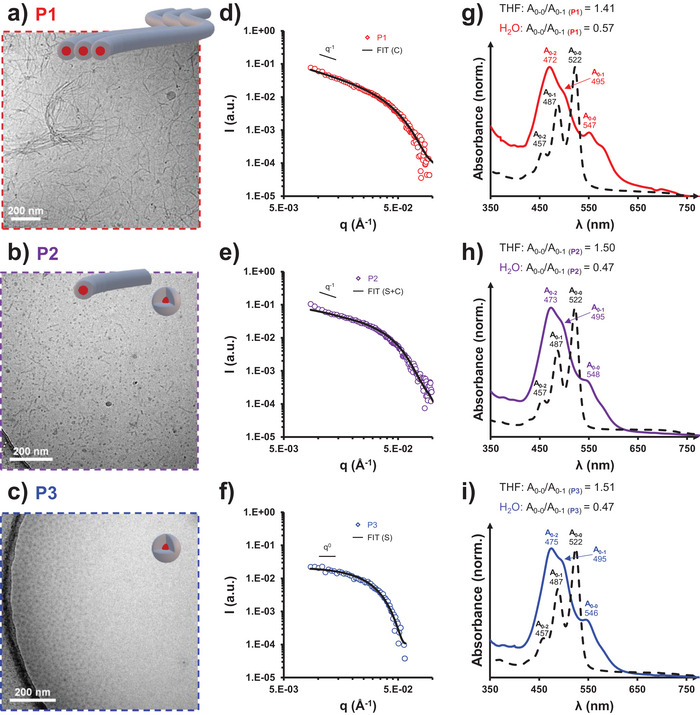
(left) Representative cryo‐TEM pictures of the 10 g/L aqueous solutions of (a) P1, (b) P2, (c) P3. The dark spots are surface contaminations stemming from water crystals. (middle) SAXS traces of (d) P1, (e) P2, (f) P3 solutions at 10 g/L in H_2_O. The black lines are models fitting the data (details shown in Table 1). (right) Normalized UV/Vis absorbance spectra of (g) P1, (h) P2, (i) P3 at 0.1 g/L in THF (black, dashed lines) and in H_2_O (color, solid line).

The aqueous polymer solutions were first analyzed by cryo‐TEM (Figure 2a–c). For **P1**, a great majority of 1D cylindrical objects were observed, with some of them forming bundles (Figure 2a). In some regions of the TEM grid, a few isolated spherical objects, supposedly vesicles, were observed in addition to the cylindrical assemblies (Figure S16). The SAXS data could be well fitted with a simple cylinder model, confirming a majority of cylindrical assemblies in sample **P1** (Figure 2d and Table 1). The increase of *DP*_n_ to 24 (sample **P2**) resulted in a mixture of short cylinders and spheres (Figure 2b). Simple sphere or cylinder models were not sufficient to fit the data over the whole q range (Figure S17), hence a model combining sphere and cylinder models was used (Figure 2e). The high proportion of spheres observed by cryo‐TEM was confirmed by SAXS (> 60% of spheres), using this model (Table 1). For both **P1** and **P2**, the upturn at low q values suggests the presence of larger aggregates. Finally, for sample **P3** with the longest polymer chain (*DP*_n_ = 134), only spherical objects with a diameter close to 15 nm were observed by cryo‐TEM and confirmed by SAXS data (Figure 2c,f). The loss of anisotropic morphologies is expected when SSDUs are combined with high molar mass polymers, as longer and bulkier polymer chains can override directional supramolecular interactions due to steric hindrance effects [55, 57, 58].

UV/Vis spectroscopy was also used as a tool to probe the PDI aggregation within the nanostructures (Figure 2g–i). Both the PDI and the polymer are soluble in THF, and the spectra obtained correspond to the fully dissolved PDI unimers, with an A_0‐0_/A_0‐1_ ratio around 1.5, typically observed for aggregate‐free PDI samples [43, 59]. In water, however, a clear change could be observed for all polymers due to the PDI aggregation. The spectra obtained are typical of co‐facially stacked PDIs, or H‐aggregates, with an A_0‐0_/A_0‐1_ ratio below 0.7 [59, 60]. The presence of these H‐aggregates was expected, as we observed by cryo‐TEM and SAXS that PDI‐PDMAcs form cylinders or spheres in H_2_O [54]. Almost no change in the A_0‐0_/A_0‐1_ ratio was noticed over time, demonstrating the strength of the PDI‐driven aggregation in water (Figure S18).

Although UV/Vis experiments reveal that the PDI sticker forms H‐aggregates in all cases, the morphology of the assemblies strongly depends on the polymer *DP*_n_. When the polymer chain is short, 1D‐nanostructures are formed in the aqueous media, but they are not colloidally stable over time, and as a result, bundles of cylinders are observed. By increasing the *DP*_n_, secondary aggregation is prevented, and the nano‐objects remain individual. However, presumably due to steric hindrance, the formation of 1D‐assemblies only is hampered, since spheres are also present. These results suggest that the PDI sticker is too hydrophobic, and the single polymer chains grafted, regardless of their length, are not able to drive the formation of long, colloidally stable nanocylinders in water.

### 2.2 Synthesis and Self‐Assembly of PDI‐TEG_2_ End‐Capped poly(*N,N*‐Dimethylacrylamide), PDI‐TEG_2_‐PDMAc

To increase the solubility and colloidal stability of the PDI‐PDMAc assemblies, two hydrophilic and flexible tri(ethylene glycol) monomethyl ether units (MeTEG) were grafted to the bay positions of the PDI. A new monofunctional perylene diimide RAFT agent, named **PDI‐TEG_2_‐TTC**, was synthesized in eight steps (Scheme S2). All procedures and characterizations are reported in the Supporting Information section (Figures S1, S6–S12d–f). The structure is similar to the **PDI‐TTC** and differs only by the two MeTEG units located at the bay positions. The bay‐functionalization leads to a slight distortion of the planar, aromatic core of the PDI as reported in the literature [56]. According to DFT calculations on simplified structures of the RAFT agents, a PDI functionalized with two ethylene glycols at the bay‐position displays a dihedral angle of 15° compared to the planar, unsubstituted PDI (Figure S13). We expect that this small distortion induced by the TEG groups should maintain the *π–π* stacking between the PDIs, while the TEG groups should help to stabilize the PDI assemblies individually in water and avoid the aggregation of the cylinders formed (bundling).

**SCHEME 2:**
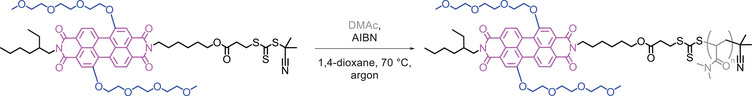
Synthesis of PDI‐TEG_2_ end‐capped PDMAc by RAFT‐mediated polymerization in the presence of PDI‐TEG_2_‐TTC.

The **PDI‐TEG_2_‐TTC** was then used for the synthesis of a series of PDI‐TEG_2_ functional PDMAcs (Scheme 2, procedure in the SI). Three polymers, with *DP*_n_ comparable to the (TEG‐free) PDI‐PDMAcs, were prepared via RAFT polymerization under the same conditions (Table 2 and Figure S1). The final polymers were characterized by SEC and ^1^H NMR (Figures S14 and S15). The SEC results showed a quantitative incorporation of the PDI‐TEG_2_ unit into the polymer chain (UV/Vis detector at 560 nm), suggesting good control of the polymerizations.

**TABLE 2:** Characteristics of the PDI‐TEG_2_ end‐capped PDMAcs: Chemical characterization and results for their self‐assembly in water.

Entry	*DP*_n, NMR_ ^a^	*M*_n, NMR_ ^a^ (kg/mol)	*M*_n, SEC_ ^b^ (kg/mol)	*Đ* ^b^	Morpho. Cryo‐TEM^c^	D_cryo‐TEM_ ^c^ (nm)	Morpho. SAXS^d^	D_SAXS_ ^d^ (nm)	L_SAXS_ ^d^ (nm)
P1‐TEG	15	2.6	2.4	1.20	Cylinders	7.8 ± 1.1	Cylinders	8.2 (0.15)	> 100 (0)
P2‐TEG	24	3.6	3.4	1.19	Cylinders	8.1 ± 1.3	Cylinders	8.6 (0.24)	36 (0)
P3‐TEG	120	13.1	8.6	1.16	Spheres	*n.d*.	Spheres	13 (0.20)	*n.d*.

^a^ Number‐average degree of polymerization, *DP*_n_, and number average molar mass, *M*_n_, of the purified polymers determined by ^1^H NMR in CDCl_3_.

^b^ *M*_n_ and dispersity, *Đ*, of the purified polymers determined by SEC in DMF (+ LiBr 1 g/L) using PMMA standards.

^c^ Morphology of the nano‐objects identified on cryo‐TEM pictures (Figure 3a–c), with the diameters of the nano‐objects and standard deviations determined using imageJ from cryo‐TEM pictures, with at least 30 measurements.

^d^ SasView models used to fit the SAXS experimental data (Figure 3d–f), with the diameter (D) and length (L) of the nano‐objects determined by fitting data, with lognormal polydispersity in parentheses.

^*^ (0) means that no polydispersity was considered in the fitting.

All polymers dissolved spontaneously at 10 g/L in H_2_O in less than 5 min, except for the **P1‐TEG**, which, visually, took 15 min to dissolve. All PDI‐TEG_2_‐PDMAcs remained colloidally stable over time (no precipitate observed).

**FIGURE 3:**
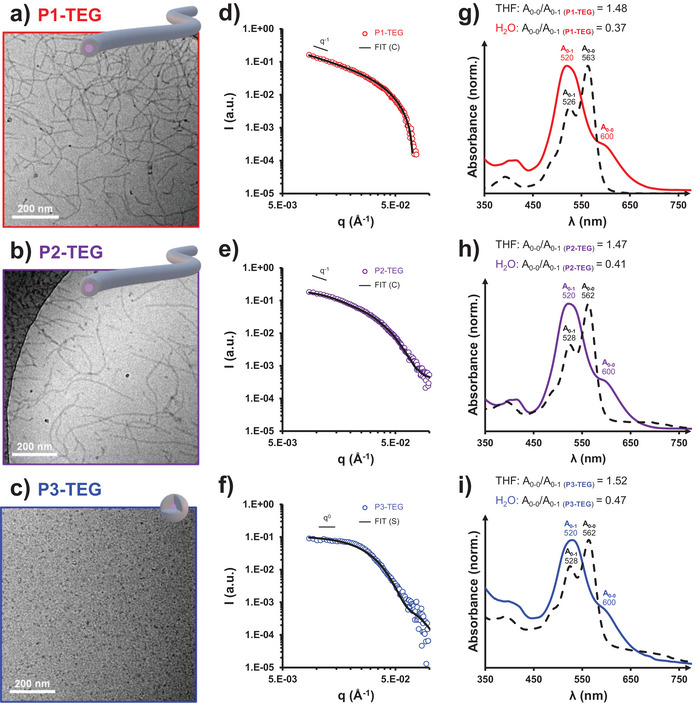
(left) Representative cryo‐TEM pictures of the 10 g/L aqueous solutions of (a) P1‐TEG, (b) P2‐TEG, (c) P3‐TEG. The dark spots are surface contaminations stemming from water crystals. (middle) SAXS traces of (d) P1‐TEG, (e) P2‐TEG, (f) P3‐TEG solutions at 10 g/L in H_2_O. The black lines are models fitting the data (details shown in Table 2). (right) Normalized UV/Vis absorbance spectra of (g) P1‐TEG, (h) P2‐TEG, (i) P3‐TEG at 0.1 g/L in THF (black, dashed lines) and in H_2_O (color, solid line).

Cryo‐TEM analyses were performed on these aqueous polymer solutions (Figure 3a–c). Pure cylinders with a diameter of *ca*. 8 nm are observed for **P1‐TEG** and **P2‐TEG**, having a *DP*_n_ 15 and 24, respectively (Figure 3a,b and Table 2). When the *DP*_n_ is further increased, only small sphere‐like objects were obtained for **P3‐TEG** (Figure 3c). SAXS confirmed the morphologies observed by cryo‐TEM (Figure 3d–f). **P1‐TEG** and **P2‐TEG** showed a clear q^−1^ decay for low q values and were well‐fitted with a cylinder model, with diameters close to those determined from cryo‐TEM images (Table 2). A sphere model was used to fit the data of **P3‐TEG** and confirmed the small size of the aggregates (13 nm).

Compared to their TEG‐free analogues, the UV/Vis absorbance spectra of the PDI‐TEG_2_‐PDMAc showed distinct spectral features in THF (Figure S19). First, due to the bay‐functionalization, the absorption maximum is slightly red‐shifted, by 40 nm (522 to 562 nm), and the signals are broader. Differences could also be observed when the spectra were recorded in water (Figure 2g–i and Figure 3g–i). As expected, UV/Vis spectroscopy measurements indicated the presence of H‐aggregates in water (Figure 3g–i). Compared to THF, where the A_0‐0_/A_0‐1_ ratio was again close to 1.5 (unimers), the A_0‐0_/A_0‐1_ ratio was around 0.4 in H_2_O, which once again confirms the aggregation of PDI [59, 60]. This ratio was also constant over time, suggesting no significant change in aggregation (Figure S20).

We can conclude that the hydrophilic MeTEG units introduced at the bay positions of the PDI effectively improved the colloidal stability of the assemblies in water: no bundles of cylinders were observed, while *π–π* stacking is still efficient and responsible for the 1D assembly in water. Overall, the results show that both PDI‐TEG_2_‐PDMAc and PDI‐PDMAc, assemblies in H_2_O are driven by H‐aggregates of PDI; for similar low *DP*_n_ (≤ 24), the formation of nanocylinders is favored in the case of PDI‐TEG_2_‐PDMAc.

**FIGURE 4:**
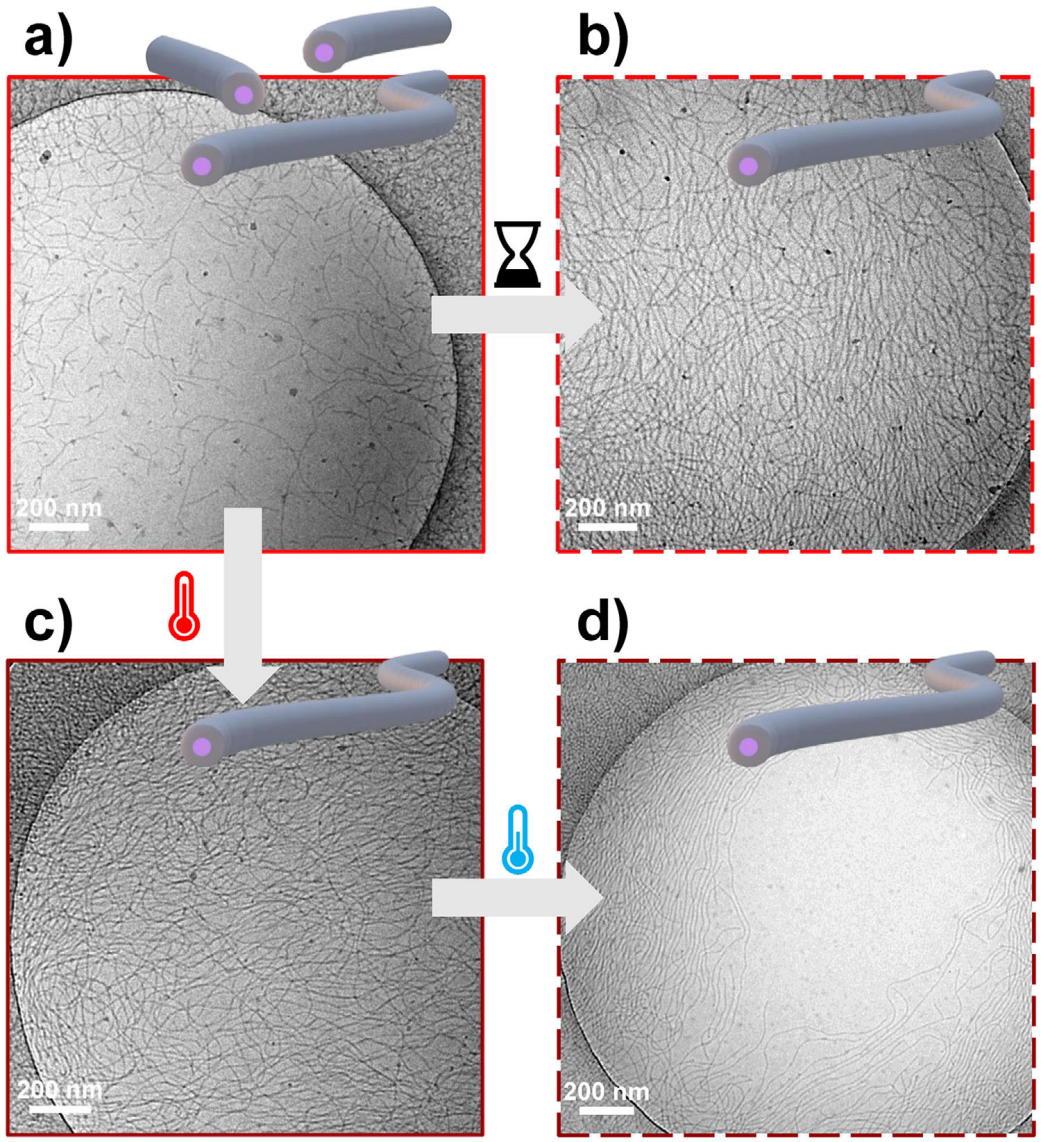
Representative cryo‐TEM pictures of the 10 g/L aqueous solutions of P1‐TEG (a) 1 day and (b) 10 months after dissolution, (c) after heating for 10 min at 60°C t (after 1 day after dissolution), and (d) 2 months after heating the solution. The dark spots are surface contaminations stemming from water crystals.

To confirm the stability of the PDI‐TEG_2_‐PDMAc nanocylinders and the absence of bundling over time, cryo‐TEM analysis was again performed 10 months after dissolution of **P1‐TEG** in water (Figure 4a,b). The cryo‐TEM images showed the presence of individual, non‐aggregated, but considerably longer cylinders with similar diameters to the initial sample (Table S2). We also found that this unexpected effect can be accelerated by thermal treatment. Heating the freshly dissolved 10 g/L solution for only 10 min at 60°C is sufficient to drive the formation of micrometer‐long cylinders (Figure 4c, Table S2, Figure S21). Visually, this transition is accompanied by an increase in the viscosity. The relative viscosity of a 3 g/L solution of **P1‐TEG** was determined using capillary viscometry and showed a large increase above 60°C (Figure S22). Interestingly, we can see by cryo‐TEM that the long nanocylinders maintain their length and diameter even after cooling down from 60°C and storing for two months (Figure 4d and Table S2). However, no significant changes could be observed by UV/Vis spectroscopy upon heating to 60°C (Figure S23). The low proportion of additional H‐aggregates formed upon heating could explain this observation.

An increase in the length of the nanocylinders upon heating was also observed for **P2‐TEG**, despite the presence of some spheres among a majority of cylinders (Figure S24 and Table S3). These experiments were also conducted on the (TEG‐free) PDI‐PDMAcs; however, no clear changes were observed after the thermal treatment (Figure S25). For **P1**, the presence of the bundles of cylinders and a few vesicular aggregates could still be observed (Figure S25a,b), while **P2** was again containing a mixture of spheres and cylinders (Figure S25c,d). These observations for the **P1‐TEG** system suggest that a thermodynamically more stable structure (long cylinders) is formed over a prolonged period after dissolution, and that the kinetics of this process can be accelerated by heating. Also, it highlights the key role that the MeTEG functionalization plays for low *DP*_n_. It allows the controlled formation of micrometer‐long nanocylinders, compared to their TEG‐free analogue, which formed a majority of bundled cylinders.

**FIGURE 5:**
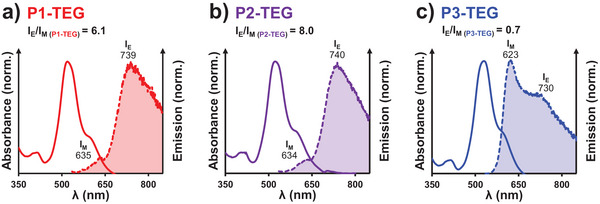
Normalized absorbance (solid lines) and fluorescence emission (dashed lines) spectra at 7.5 10^−6^ mol/L in H_2_O of (a) P1‐TEG, (b) P2‐TEG, (c) P3‐TEG. The small insert at the top of the spectra shows the ratio between excimer (E) and monomer (M) emission intensity. λ_excitation_ was set at 520 nm. It is important to compare these fluorescence spectra at the same molar concentration, as the I_E_/I_M_ ratio decreases with concentration (Figure S26).

Finally, to study more deeply the PDI aggregates, fluorescence spectra were recorded in water (Figure 5). In THF (Figure S19), the absorbance (λ_max_ = 562 nm) and emission (λ_max_ = 588 nmvis, Stokes shift = 1360 cm^−1^) spectra were characteristic of the isolated unimers in solution. Meanwhile, in water, larger Stokes shifts were observed, and the spectra were no longer mirror images of each other, giving us an indication that the emission signal was greatly affected by the PDI aggregation. Various examples of 1D systems, where the conjugated π‐stacked cores are co‐facially aligned, show that an excimer (excited‐state dimer) or excimer‐like species can be observed by fluorescence [52]. The spectral signature of this excimer species is characterized by a broad emission band, greatly red‐shifted compared to the monomer/unimer emission [61]. The fluorescence spectra obtained for the PDI‐TEG_2_‐PDMAc in water are quite different from those obtained in THF (Figure S19b), and display two bands. The first band, at 630 nm (Stokes shift = 3,500 cm^−1^), is attributed to the monomer emission, whereas the red‐shifted band at 740 nm (Stokes shift = 5,700 cm^−1^) could be attributed to the excimer emission [20, 62]. An I_E_/I_M_ ratio was used to quantify the excimer emission, similarly to other systems using a pyrene probe [63, 64]. For **P1‐TEG** and **P2‐TEG,** which form cylinders, the ratio was above 6, whereas for **P3‐TEG**, which contains spheres, this ratio decreased to 0.7. This suggests that the PDIs are organized differently, supposedly closer, with stronger aggregation within the (1D) cylinders compared to the spheres. As detailed in the Supporting Information, similar results were obtained for the (TEG‐free) PDI‐PDMAcs, which confirm this hypothesis (Figure S27).

## 3 Conclusion

In summary, we have demonstrated that long, co‐facially stacked 1D nanocylinders can be formed using PDI‐functional PDMAcs by direct dissolution in water. We studied two analogous systems that differ only by the presence of two MeTEG units at the bay‐position of the PDI sticker. Cryo‐TEM and SAXS analyses of the aqueous polymer solutions showed the formation of cylinders for the lowest *DP*_n_, whereas spheres were formed when increasing the polymer chain length. This study reveals the importance of MeTEG functionalization of the PDI sticker, as without MeTEG, the cylinders obtained are not colloidally stable and tend to bundle. We suggest that the presence of TEG protects the PDI core from lateral hydrophobic aggregation and facilitates controlled assembly. For all the nanostructures observed, UV/Vis spectroscopy helped us revealing the presence of H‐aggregates. For the PDI‐TEG_2_‐PDMAc, long (micrometric) cylinders formed upon aging of the aqueous solution or via thermal treatment. This transition was accompanied by a threefold increase in relative viscosity, associated with the formation of longer nano‐objects.

Finally, fluorescence emission spectroscopy revealed the formation of excimer‐like species in water, due to the strong *π–π* interactions of the PDI units. Higher excimer emission in the cylinders was observed compared to the spheres, suggesting better organization and co‐facial alignment in the cylinders.

This work demonstrated a simple and solvent‐free method to obtain long‐range 1D nanostructures based on PDI. This introduces new structuration and processing techniques of organic π‐conjugated materials for optoelectronic applications.

## Author Contributions

J.R., L.S.V., and S.B. contributed to the conceptualization of the work presented in the manuscript. J.M.G. and S.B. were responsible for data curation, with C.H. contributing to SAXS analysis and J.P.C. to DFT analysis as part of the formal data analysis. Data visualization was carried out by S.B. and L.S.V., while the investigation was conducted by S.B. and J.M.G. Resources were provided by J.M.G. and C.H., for cryo‐TEM and SAXS, respectively. J.R. and L.S.V. secured funding and supervised the project. The initial draft was written by S.B. and J.R., and the final review and editing of the submitted manuscript were performed by S.B., J.R., L.S.V., L.B., and J.P.C.

## Conflicts of Interest

The authors declare no conflicts of interest.

## Supporting information




**Supporting Figures and Tables**: marc70015‐sup‐0001‐SuppMat.pdf

## Data Availability

The data that support the findings of this study are available in the supplementary material of this article.
