# Supplementary material for: Direct Assembly of Micrometer‐Long Polymeric Cylinders in Water via Supramolecular Sticker Engineering
Source: Macromol Rapid Commun. 2025 Aug 14;46(21):e00478. doi: 10.1002/marc.202500478 (PMC12590929; doi:10.1002/marc.202500478)
Supplement: Supplementary file 1 — Supporting Figures and Tables: marc70015‐sup‐0001‐SuppMat.pdf [file MARC-46-e00478-s001.pdf]

Supporting Information

Direct assembly of micrometer-long polymeric cylinders in water via supramolecular sticker engineering

Sébastien Berruée[a], Jean-Michel Guigner[b], Cécile Huin[a, c], Jan Patrick Calupitan[a], Laurent Bouteiller[a], Lydia Sosa Vargas*[a], Jutta Rieger*[a]

[a] Sorbonne Université, CNRS, Institut Parisien de Chimie Moléculaire (IPCM), F-75005, Paris, France

[b] Sorbonne Université, CNRS, Institut de Minéralogie, de Physique des Matériaux et de Cosmochimie (IMPMC), F-75005 Paris, France

[c] Université Evry Paris-Saclay, 91000 Evry, France

E-mail : lydia.sosa-vargas@sorbonne-universite.fr; jutta.rieger@sorbonne-universite.fr

Table of contents

1. Materials 2
2. Characterizations 3
3. Synthetic procedures 5
4. Self-assembly in water 32
5. References 42

1. Materials

3,4,9,10-Perylenetetracarboxylic dianhydride (PDA, >98%, TCI), *n*-butanol (*n*-BuOH, >99%, TCI), butyl bromine (BuBr, 99% ACROS Organics), 1,8-diazabicyclo[5.4.0]undec-7-ene (DBU, 98%, Aldrich), bromine (Br_2, Aldrich), potassium carbonate (K_2CO_3, ≥ 99%, Honey well), sodium thiosulfate ($Na_2S_2O_3$, >98%, Fischer Sci.), *p*-toluenesulfonic acid monohydrate (*p*-TsOH.H_2O, ≥ 99%, Sigma-Aldrich), 2-ethylhexylamine (98%, Aldrich), glacial acetic acid (AcOH, 99.7%, Fischer Sci.), 6-aminohexan-1-ol (95%, abcr), sodium hydride (NaH, 60% dispersion in mineral oil, Aldrich), thionyl chloride ($SOCl_2$, >98%, Aldrich) were used as received.

Tri(ethylene glycol) monomethyl ether (MeTEG, 95%, Aldrich) was dried by three successive azeotropic distillations with dry toluene and kept on 4 Å molecular sieves under an argon atmosphere. *N,N-D*imethylacrylamide (DMAc, >99%, Aldrich) was distilled before use. 1,4-Dioxane (>99%, TCI) was passed through basic alumina to remove the BHT inhibitor. 2,2'-Azobis(2-methylpropionitrile) (AIBN, ≥ 98%, Aldrich) was recrystallized in methanol.

Anhydrous dichloromethane (DCM) and tetrahydrofuran (THF) were obtained from a MBraun solvent purification system (MB SPS-800) whereas, acetonitrile (ACN, 99.9%, VWR, Normapur), chloroform ($CHCl_3$, 99.8%, VWR Chemicals), dichloromethane (DCM, 99%, Fischer Sci.), *n*-heptane (99.8%, VWR Chemicals), toluene (VWR Chemicals), tetrahydrofuran (THF, 100%, VWR Chemicals), methanol (MeOH, >99%, Carlo Erba), ethanol (EtOH, Carlo Erba), *n*-pentane (98%, Aldrich), *N,N*-dimethylformamide (DMF, extra dry 99.8%, ACROS Organics), diethyl ether (Et_2O, VWR Chemicals) were used as received for the reactions and the purification steps. All aqueous solutions and treatments were prepared with deionized water. All polymerizations and some syntheses (as indicated) were performed under an argon atmosphere.

Heating of most of the reactions was done using heating blocks, except for the polymerizations, where an oil bath was used. When temperature control was needed, an inner glass-covered probe (thermometer) was employed. Reactions performed at room temperature (RT) were done at temperature ranges of 20-22 °C. Thin layer chromatography (TLC silica gel 60 F254, 2x4cm plates, Merck) was used to follow the reaction progress, and the consumption of the starting material by using an eluent mixture of $CHCl_3$/MeOH (96/4, V/V) unless indicated otherwise. After extractions, the organic phase was dried with anhydrous magnesium sulfate ($MgSO_4$, VWR Chemical). Reagents were dried at 70°C using Dry-Line oven from VWR Chemicals or under vacuum using a Vacuubrand GMBH before further reaction.

The purifications performed by chromatography were either carried out by silica plug or by column chromatography using silica-gel as the stationary phase (Si 60, 40-63 µm, Merck®), or by flash chromatography using a Bucchi® Pure C-805 Flash with pre-packed Bucchi FlashPure EcoFlex® silica columns (40 g).

2. Characterizations

Nuclear Magnetic Resonance spectroscopy (NMR)

The identity of the target molecules was determined by ^{1}H and ^{13}C NMR. Spectra were recorded at 298 K, with a Bruker 300 or 400 MHz spectrometer in 5 mm diameter tubes. Deuterated chloroform ($CDCl_3$, 99.8%, Eurisotop), deuterium oxide (D_2O, 99.96%, Eurisotop), acetone-d6 (99.8%, Eurisotop) were used as NMR solvents. Monomer conversions were determined by ^{1}H NMR spectroscopy in $CDCl_3$ by the relative integration of the internal reference (DMF signal at 7.9 ppm) and the vinylic monomer proton signals.

Size-Exclusion Chromatography (SEC)

SEC analyses were carried out on two PSS GRAM 1000 Å columns (8 × 300 mm; separation limits: 1 to 1000 kg/mol) and one PSS GRAM 30 Å column (8 × 300 mm; separation limits: 0.1 to 10 kg/mol) coupled with a differential refractive index detector (RI) and a UV detector (λ = 560 nm, maximal absorption of the perylene diimide unit). DMF (+ LiBr 1 g/L) at 60 °C was used as the mobile phase at a flow rate of 0.8 mL/min. Samples were prepared at a concentration of 2.5 mg/mL and filtered through a 0.20 µm pore-size PTFE membrane before the injection (V_{inj} = 100 µL). The number-average molar mass (M_n), the weight-average molar mass (M_w), and the molar mass dispersity ($Đ = M_w/M_n$) were calculated from the RI signals with OmniSEC 5.12 software thanks to a conventional calibration curve based on PMMA standards.

Cryo-Transmission Electron Microscopy (cryo-TEM)

The samples were prepared a few days (between 1 and 11 days) before analysis, unless mentioned otherwise. 3 µL of the aqueous polymer solutions (at 10 g/L) were deposited on a quantifoil grid. The excess of solution was removed with a Whatman paper, and the grid was immediately frozen in liquid ethane. The observations were carried out at -180 °C by a JEOL JEM-2100 LaB_6 microscope operating at 200 kV. The images were taken on a Gatan US1000, 2k x 2k CCD Camera. Dimensions (height or thickness) were determined from cryo-TEM images using

the software ImageJ (https://imagej.net/ij/), using at least 30 points. The mean value and standard deviation were determined from these data.

Small-Angle X-ray Scattering (SAXS)

Small-Angle X-ray Scattering experiments were performed on a Nano-inXider SW system (Xenocs) in transmission mode using Cu Kα radiation (λ = 1.54 Å, 8 keV) from an X-ray microsource (GeniX3D) operating at 50 kV-0.6 mA (30 W). Scattering patterns were collected using an in-vacuum 2D Pilatus3 Hybrid pixel detector (83.8 x 33.5 mm^2, pixel size: 172 x 172 μm^2) (Dectris), localized at a distance of 938 mm. The collimation was chosen to maximize the intensity while adjusting the *q* range to the studied samples. The *q* range is between 0.00859 $Å^{-1}$ and 0.1 $Å^{-1}$. The reduction of 2D datasets from the Pilatus3 detector to 1D was achieved by radially averaging the raw data detector counts using Xsact data reduction software. Then, SAXS spectra were normalized by the acquisition time and by the number of transmitted photons.

The measurements were performed at a concentration of 10 g/L. Standard correction procedures were applied for X-ray beam transmission, detector efficiency, and signal subtraction of the 1 mm capillary filled with the solvent. The data were fitted using the SasView software (http://www.sasview.org/). According to the observed morphologies using cryo-TEM, the data were fitted with different models.

Ultraviolet-visible (UV/Vis) absorption spectroscopy

The absorbance spectra at 25 °C were recorded with a Jasco V-670 spectrometer. Analyses were performed in 10 x 10 mm or 10 x 2 mm quartz cuvettes. Analyses in THF were done at a scan rate of 400 nm/min, from 750 to 350 nm. The spectra acquired in aqueous solutions were carried out at a scan rate of 200 nm/min, from 850 to 350 nm. 50 or 100 scans were performed for the most diluted samples. All solutions were prepared by diluting the 10 g/L stock solution with H_2O. The baseline (recorded with the pure solvent) was removed from the absorbance values obtained.

Fluorescence emission spectroscopy

The fluorescence emission spectra were measured using a FluoroMax-4 (Horiba Jobin Yvon) spectrofluorometer. Analyses were performed in 10 mm x 10 mm quartz cuvettes on diluted solutions (concentrations of the solutions are indicated for each analysis). The excitation wavelength was set at 490 nm and 520 nm for PDI-PDMAc and PDI-TEG_2-PDMAc, respectively. The integration time was set at 0.1 s for all spectra.

Capillary viscometry

Analyses were performed with 0.6 mL of solution using a Cannon-Manning Semi-Micro viscometer. Heating was performed using a water bath equipped with a temperature control. Experiments were first recorded with pure water, and then with the polymer solution (of **P1-TEG**) at 3 g/L. The samples were filtered with a 0.5 µm PTFE filter and equilibrated for 5 min at the desired temperature before analyses. The relative viscosity was determined for each temperature as the ratio between the flow times of the polymer solution and water.

Computational modelling – Density Function Theory (DFT)

DFT calculations were performed using the Gaussian16[1] suite of programs. The results were visualized by GaussView. Initially, the structure was optimized using the B3LYP functional[2,3] as it was shown to be effective for heterocyclic structures.[4,5] The basis set 6-31+g(d) was used. Frequency calculation on the optimized structure revealed no imaginary frequencies, verifying that the optimized structure corresponds to an absolute minimum. The results were visualized by GaussView and treated by GaussSum.[6]

3. Synthetic procedures

a) Synthesis of the PDI RAFT agents, PDI-TTC (6) and PDI-TEG$_2$-TTC (13)

The two monofunctional PDI RAFT agents were obtained *via* an esterification between a trithiocarbonate RAFT agent functionalized by a carboxylic acid (**TTC-COOH**) previously reported[7], and an alcohol-functionalized non-symmetrical PDI. Syntheses of **PDI-TTC (6)** and **PDI-TEG$_2$-TTC (13)** are depicted in **Scheme S1** and **Scheme S2**, respectively.

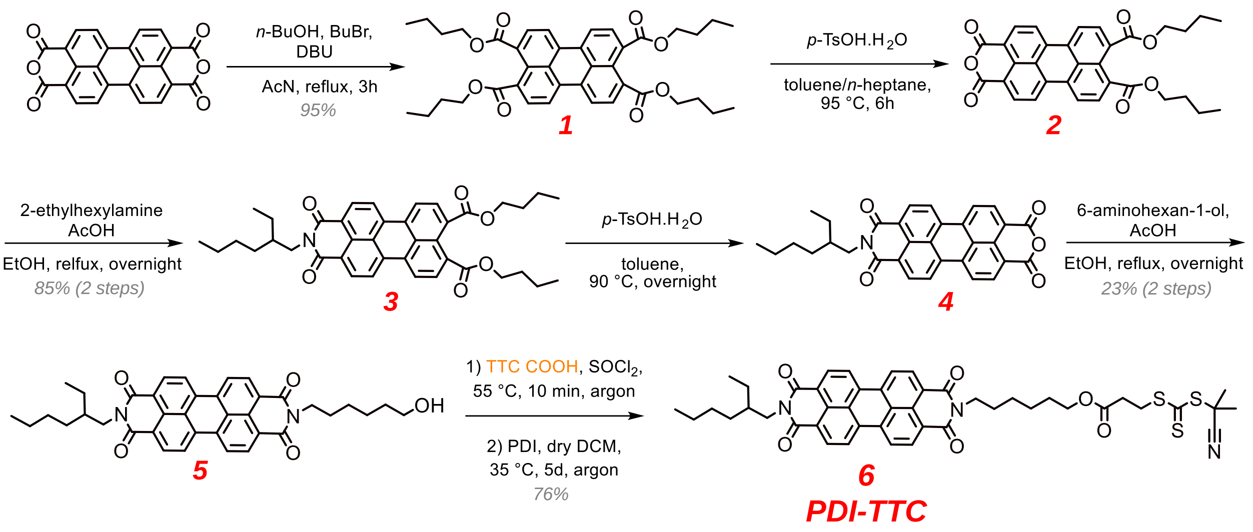

Scheme S1: Synthetic route of the monofunctional PDI RAFT agent (**PDI-TTC**).

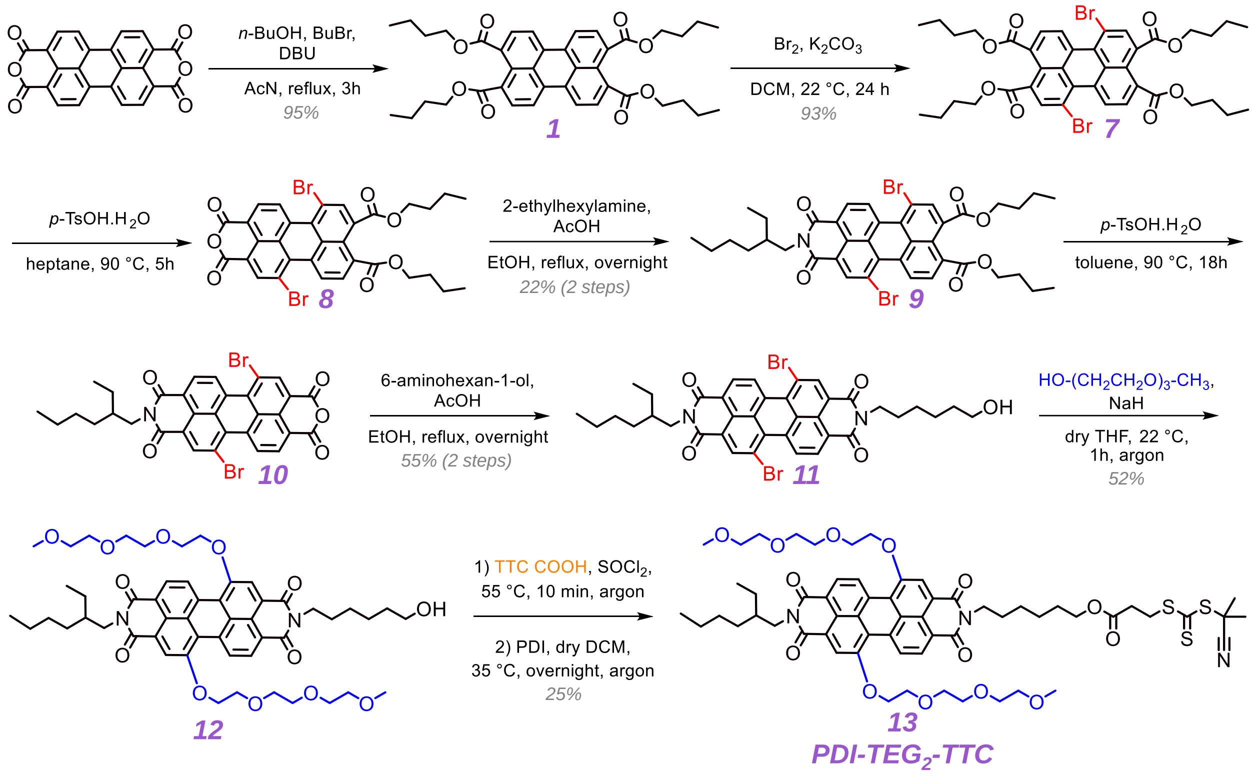

Scheme S2: Synthetic route of the monofunctional PDI RAFT agent functionalized with two MeTEG at the bay-positions (**PDI-TEG₂-TTC**).

❑ *Synthesis of PDI-TTC*

Synthesis of tetrabutyl perylene-3,4,9,10-tetracarboxylate (1, PTE)[8]

PDA (14.2 g, 36 mmol, 1 eq), *n*-butanol (27 mL, 291 mmol, 8.1 eq), 1-bromobutane (31 mL, 287 mmol, 8.0 eq) and DBU (22 mL, 147 mmol, 4.1 eq) were solubilized in 200 mL of acetonitrile and heated under reflux during 3h. The initial brownish-red suspension quickly turns into an orange solid insoluble in acetonitrile.

The solution was cooled to RT, and the bright orange solid was filtered under vacuum using a Büchner funnel. The recovered solid was washed with acetonitrile, then methanol until the dark orange filtrate (green/yellow under UV) becomes almost colorless. The resulting orange solid was dried overnight at 100 °C (22.5 g, 95%).

δ_H (300 MHz, $CDCl_3$) = 7.97 (H_A, d, 4H); 7.88 (H_B, d, 4H); 4.36 (H_C, t, 8H); 1.81 (H_D, quintet, 8H); 1.53 (H_E, sextet, 8H); 1.02 (H_F, t, 12H) ppm.

All other characterization data agree with the literature.[8]

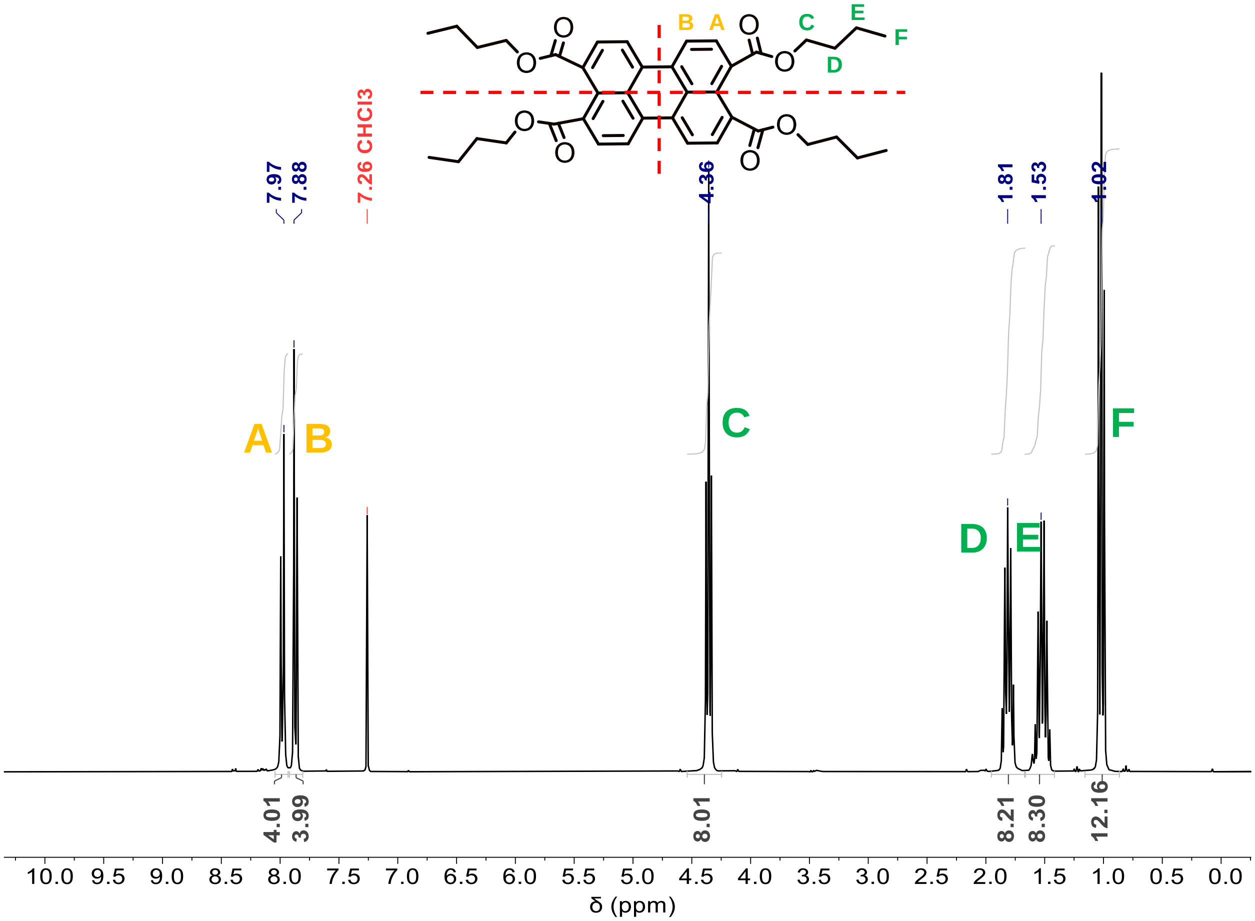

Figure S1: ^{1}H NMR of **(1)** recorded in $CDCl_3$.

Synthesis of dibutyl 1,3-dioxo-1H,3H-benzo[10,5]anthra[2,1,9-def]isochromene-8,9-dicarboxylate (2, PMADE)[8]

PTE **(1)** (8.55 g, 13 mmol, 1 eq) was added to an RBF and solubilized in 120 mL of a *n*-heptane/toluene (5/1, v/v) mixture. Then, *p*-TsOH.H_2O (2.6 g, 14 mmol, 1.04 eq) was added, and the reaction was stirred at 95 °C for 4h. A red precipitate formed over time.

The flask was cooled to RT, the solid filtered under vacuum, and washed with water and MeOH. The crude, dark red solid (6.2 g) was used directly for the next step even though it contained a mixture of symmetrical PDA, PMADE **(2),** and PTE **(1)**. The subsequent step will facilitate the separation of this mixture.

Synthesis of dibutyl 2-(2-ethylhexyl)-1,3-dioxo-2,3-dihydro-1H-benzo[10,5]anthra[2,1,9-def]isoquinoline-8,9-dicarboxylate (3, PMIDE)

The crude product from the previous step, containing PMADE **(2)** (6.2 g, 1.2 10^{-2} mol, 1 eq), 2-ethylhexyl amine (2.7 mL, 1.7 10^{-2} mol, 1.4 eq) and AcOH (0.3 mL, 5.8 10^{-3} mol, 0.5 eq) were solubilized in 175 mL of ethanol in a single-necked RBF. The mixture was stirred overnight under reflux. The product was purified using column chromatography, depositing the crude via dry loading. Toluene was first used as a solvent to remove the PTE **(1)** as an orange fraction. Then, a mixture of DCM with 2% of MeOH was used to recover the target PMIDE **(3)** as a red fraction, which, once the solvent evaporated, gave a dark red solid (7.0 g, 85 % yield after two steps).

δ_H (300 MHz, $CDCl_3$) = 8.09 (H_A, d, 2H); 7.77-7.80 (H_B, mult, 6H); 4.36 (H_C, t, 4H); 4.03 (H_D, mult, 2H); 1.92 (H_E, mult, 1H); 1.84 (H_F, quintet, 4H); 1.55 (H_G, sextet, 4H); 1.37 (H_H, mult, 8H); 1.04 (H_I, t, 6H); 0.96 (H_J, t, 3H); 0.91 (H_J, t, 3H) ppm.

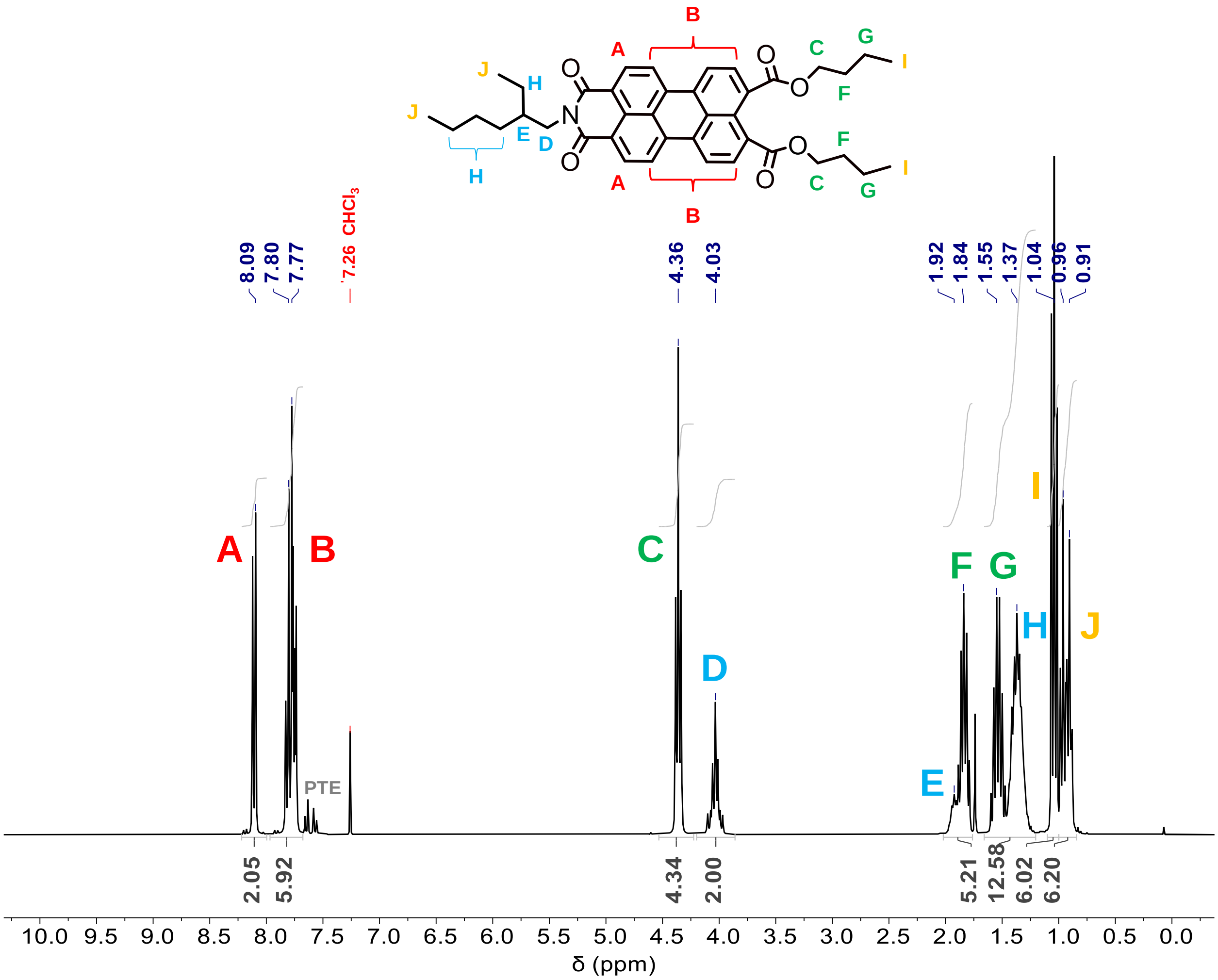

Figure S2: ^{1}H NMR of **(3)** recorded in $CDCl_3$.

Synthesis of 9-(2-ethylhexyl)-1H-isochromeno[6',5',4':10,5,6]anthra[2,1,9-def]isoquinoline-1,3,8,10(9H)-tetraone (4, PMIMA)

PMIMA **(4)** was synthesized using a modified procedure from Sengupta *et al*.[9] PMIDE **(3)** (7.0 g, 11 mmol, 1 eq) was added to an RBF and solubilized in 250 mL of toluene. Then, *p*-TsOH.H_2O (16.4 g, 86 mmol, 7.8 eq) was added, and the reaction was stirred at 90 °C for two days, during which a red precipitate formed over time.

The flask was cooled to RT, the solid filtered under vacuum, and washed with water and MeOH. The red solid (5.5 g) was used directly for the next step as it was not soluble enough for ^{1}H NMR characterization.

Synthesis of 2-(2-ethylhexyl)-9-(6-hydroxyhexyl)anthra[2,1,9-def:6,5,10-d'e'f']diisoquinoline-1,3,8,10(2H,9H)-tetraone (5)[10]

PMIMA **(4)** (5.5 g, 1.0 10^{-2} mol, 1 eq), 6-aminohexan-1-ol (1.8 g, 1.5 10^{-2} mol, 1.5 eq), and AcOH (0.3 mL, 5.2 10^{-2} mol, 0.5 eq) were solubilized in 200 mL of ethanol in a single-necked RBF. The mixture was stirred overnight under reflux. The resulting product was purified using column chromatography, depositing the crude via dry loading. DCM was first used as a solvent to remove an orange fraction corresponding to the remaining PMIDE **(3)**. Then, a mixture of DCM with 2% of MeOH was used to obtain the target non-symmetrical PDI **(5)** as an orange fraction, which after evaporation, resulted in a red solid (1.6 g, 23% yield after two steps).

δ_H (300 MHz, $CDCl_3$) = 8.59 (H_A, d, 4H); 8.51 (H_B, d, 4H); 4.20 (H_C, t, 2H); 4.13 (H_D, mult, 2H); 3.67 (H_E, t, 2H); 1.96 (H_F, mult, 1H); 1.40-1.80 (H_{G+H}, mult, 16H); 0.96 (H_I, t, 3H); 0.89 (H_I, t, 3H) ppm.

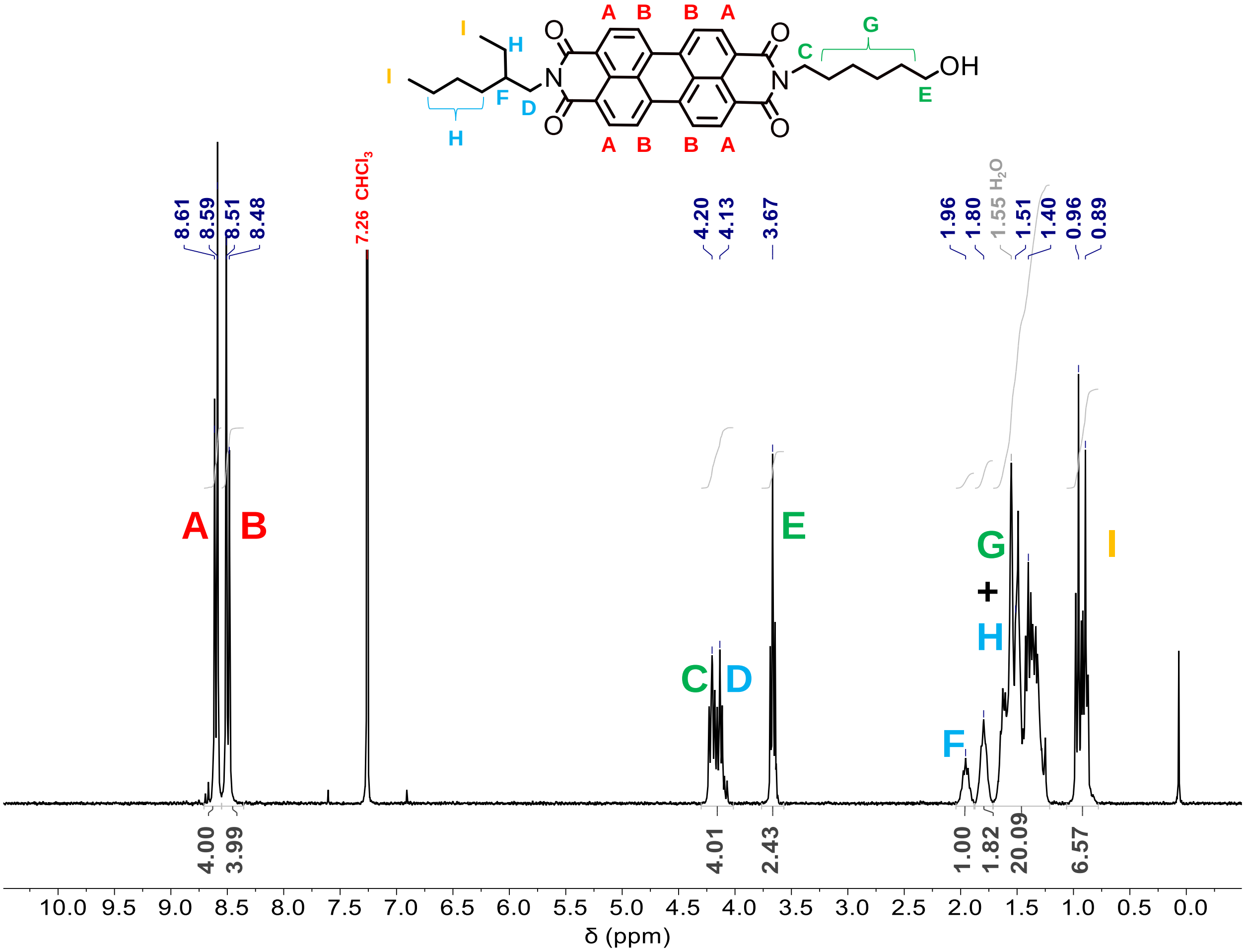

Figure S3: ^{1}H NMR of **(5)** recorded in $CDCl_3$.

Synthesis of 6-(9-(2-ethylhexyl)-1,3,8,10-tetraoxo-3,8,9,10-tetrahydroanthra[2,1,9-def:6,5,10-d'e'f']diisoquinolin-2(1H)-yl)hexyl 3-((((2-cyanopropan-2-yl)thio)carbonothioyl)-thio)propanoate (6, PDI-TTC)

PDI-TTC (6) was synthesized using a procedure from our previous work.[7] **TTC-COOH** (326 mg, 1.3 mmol, 2.5 eq) was added to a 10 mL Schlenk flask, set under argon before adding $SOCl_2$ (0.55 mL, 6.9 mmol, 13 eq) and stirring the mixture for 10 min at 55 °C to form TTC-COCl (oily liquid). The remaining $SOCl_2$ was removed under vacuum and evaporated using dry toluene (two additions). In parallel, the non-symmetrical PDI **(5)** (309 mg, 0.5 mmol, 1 eq) was added to a 100 mL RBF and solubilized in 60 mL of dry DCM under argon. Then, the TTC-COCl was dissolved in 10 mL of dry DCM and added dropwise to the PDI solution at 0 °C. The mixture was stirred for 5 days at 35 °C.

4 mL of EtOH was added to quench the excess TTC-COCl, and the mixture was stirred for 30 min before removing the solvent under reduced pressure. The product was precipitated three times in *n*-pentane to remove the free RAFT agent (TTC-COOEt). The product was purified using column chromatography, depositing the crude via dry loading. A mixture of $CHCl_3$ with 0.25% MeOH was used to recover the desired product. After solvent evaporation, the final **PDI-TTC (6)** was obtained as a red solid (328 mg, 76%).

δ_H (400 MHz, $CDCl_3$) = 8.24 (H_A, d, 4H); 8.03 (H_B, d, 4H); 4.13 (H_C, t, 2H); 4.11 (H_E, t, 2H); 4.03 (H_D, mult, 2H); 3.60 (H_J, t, 2H); 2.76 (H_K, t, 2H); 1.87 (H_F, mult, 1H); 1.87 (H_L, s, 6H); 1.36-1.77 (H_{G+H}, mult, 16H); 0.95 (H_I, t, 3H); 0.90 (H_I, t, 3H) ppm.

δ_C (100 MHz, $CDCl_3$) = 217 ($C_{C=S}$), 171 ($C_{C=O\ ester}$), 163 ($C_{C=O\ imide}$), 122-133 ($10C_{aromatic}$), 120 ($C_{nitrile}$), 65 (C_{CH2-O}), 10-44 ($17C_{alkyl}$) ppm.

HRMS (APCI) m/z: $[M+H]^+$ Calculated: ($C_{46}H_{47}N_3O_6S_3H$); 834.2627 **Found:** 834.2686

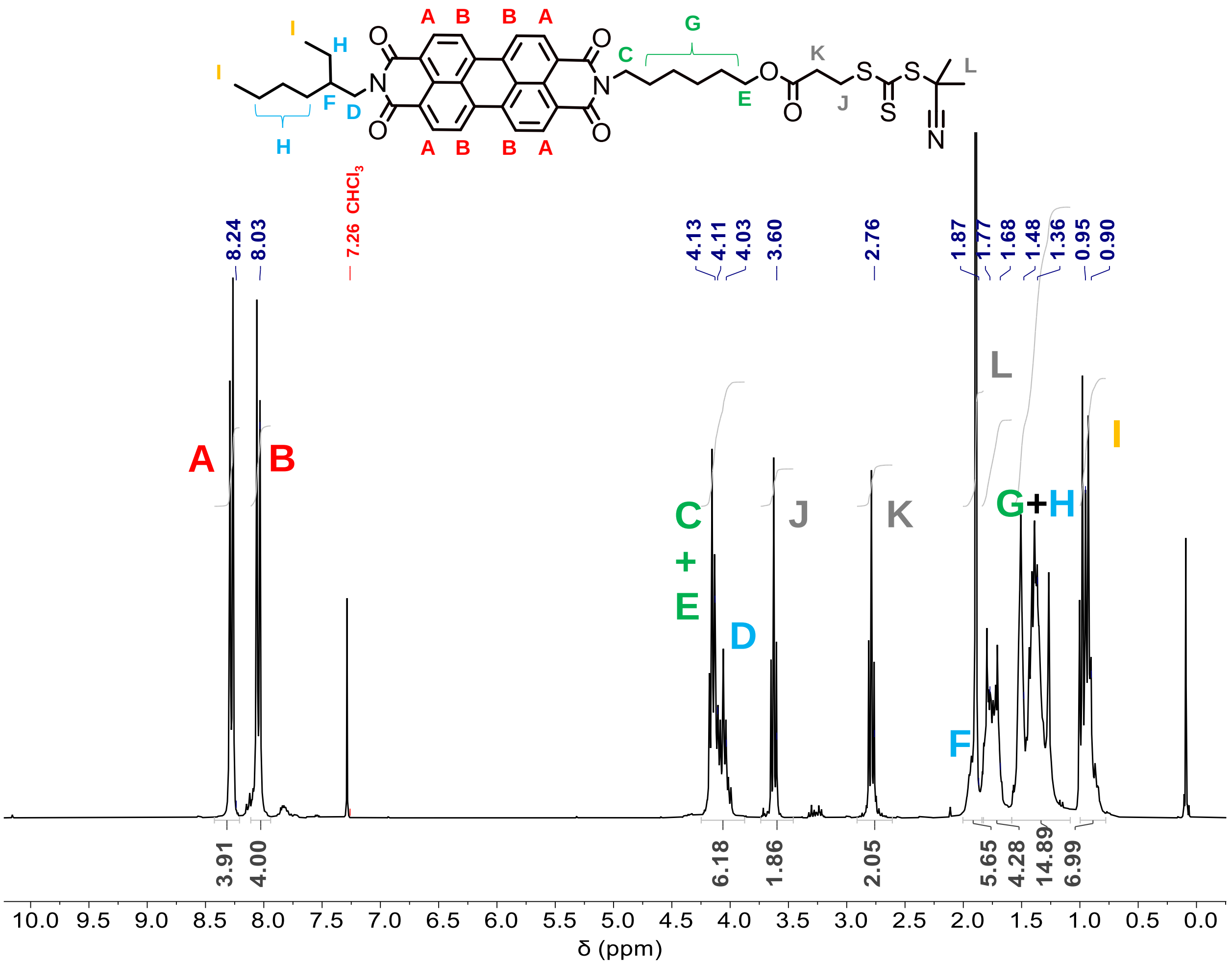

Figure S4: ^{1}H NMR of the final **PDI-TTC (6)** recorded in $CDCl_3$.

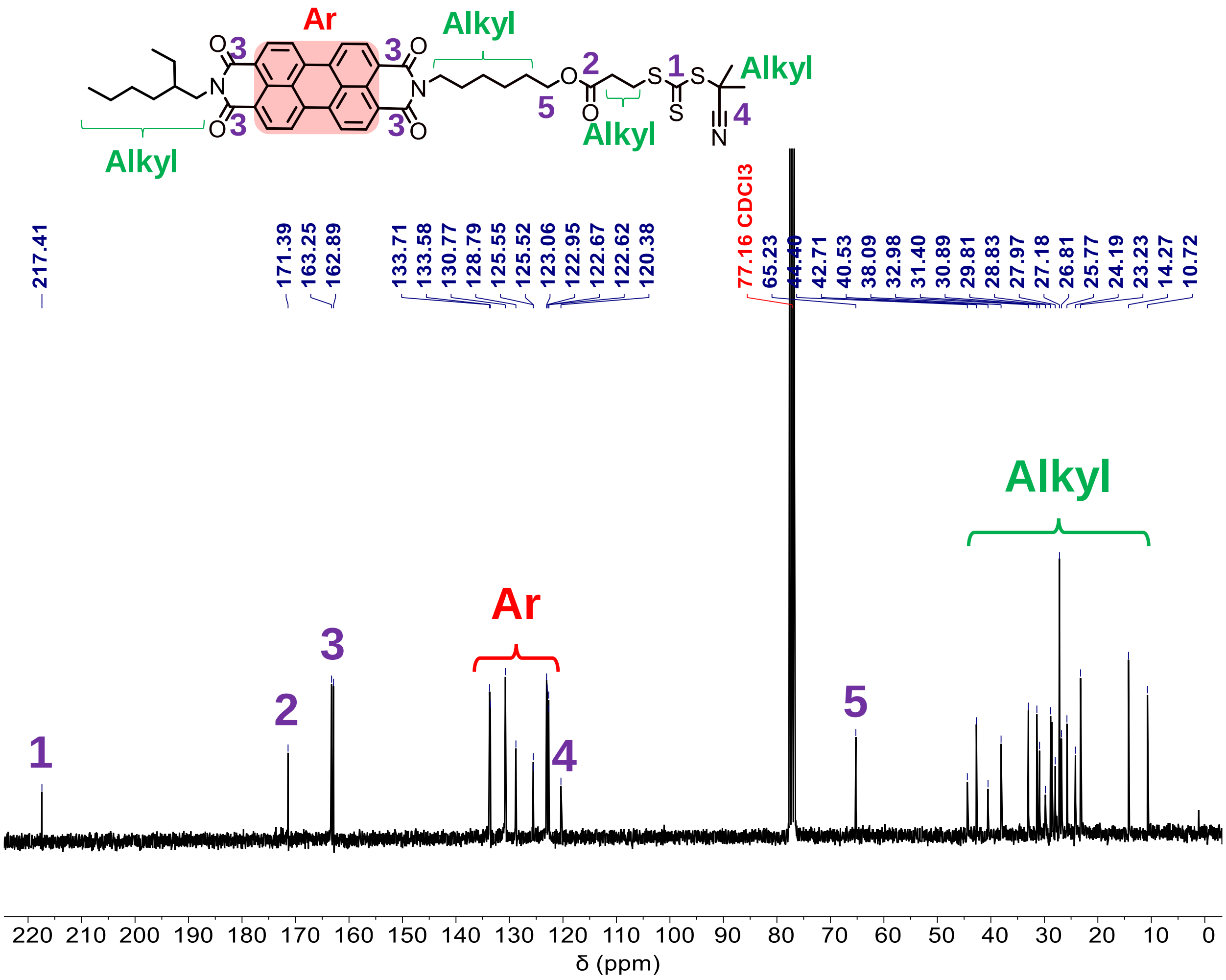

<u>Figure S5:</u> ^{13}C NMR of the final **PDI-TTC (6)** recorded in $CDCl_3$.

❑ *Synthesis of PDI-TEG₂-TTC*

Synthesis of tetrabutyl 1,7-dibromoperylene-3,4,9,10-tetracarboxylate (7, PTE Br_2)

PTE Br_2 **(7)** was synthesized using a modified procedure from Jia *et al*.[8] PTE **(1)** (9.3 g, 14 mmol, 1 eq) and K_2CO_3 (4.9 g, 35 mmol, 2.5 eq) were solubilized in 130 mL of DCM in a 2-necked RBF, resulting in an orange solution. The flask was linked to a trap containing an aqueous sodium thiosulfate solution. Then, bromine (9.4 mL, 183 mmol, 13 eq) was added dropwise over 5 min at 22 °C to the solution of **(1)** in DCM, which turned dark red/black after bromine addition. The resulting solution was stirred for 24h at 22 °C.

The reaction was then bubbled with argon to remove unreacted bromine and quenched with the trap. After 1 h, no color was observed in the trap. A saturated aqueous sodium thiosulfate solution

was then added dropwise to the solution at 0°C using an ice bath. The organic phase was then washed three times with H_2O. This dark brown organic phase was dried over $MgSO_4$ and filtered before evaporation of the solvent under reduced pressure. The product was passed through a silica plug using DCM as eluent to recover the PTE Br_2 **(7)** as an orange solid (10.8 g, 93%), which was characterized by ^{1}H NMR.

δ_H (300 MHz, $CDCl_3$) = 8.92 (H_A, d, 2H); 8.29 (H_C, s, 2H); 8.06 (H_B, d, 2H); 4.35 (H_D, t, 8H); 1.79 (H_E, quintet, 8H); 1.49 (H_F, sextet, 8H); 1.00 (H_G, t, 12H) ppm.

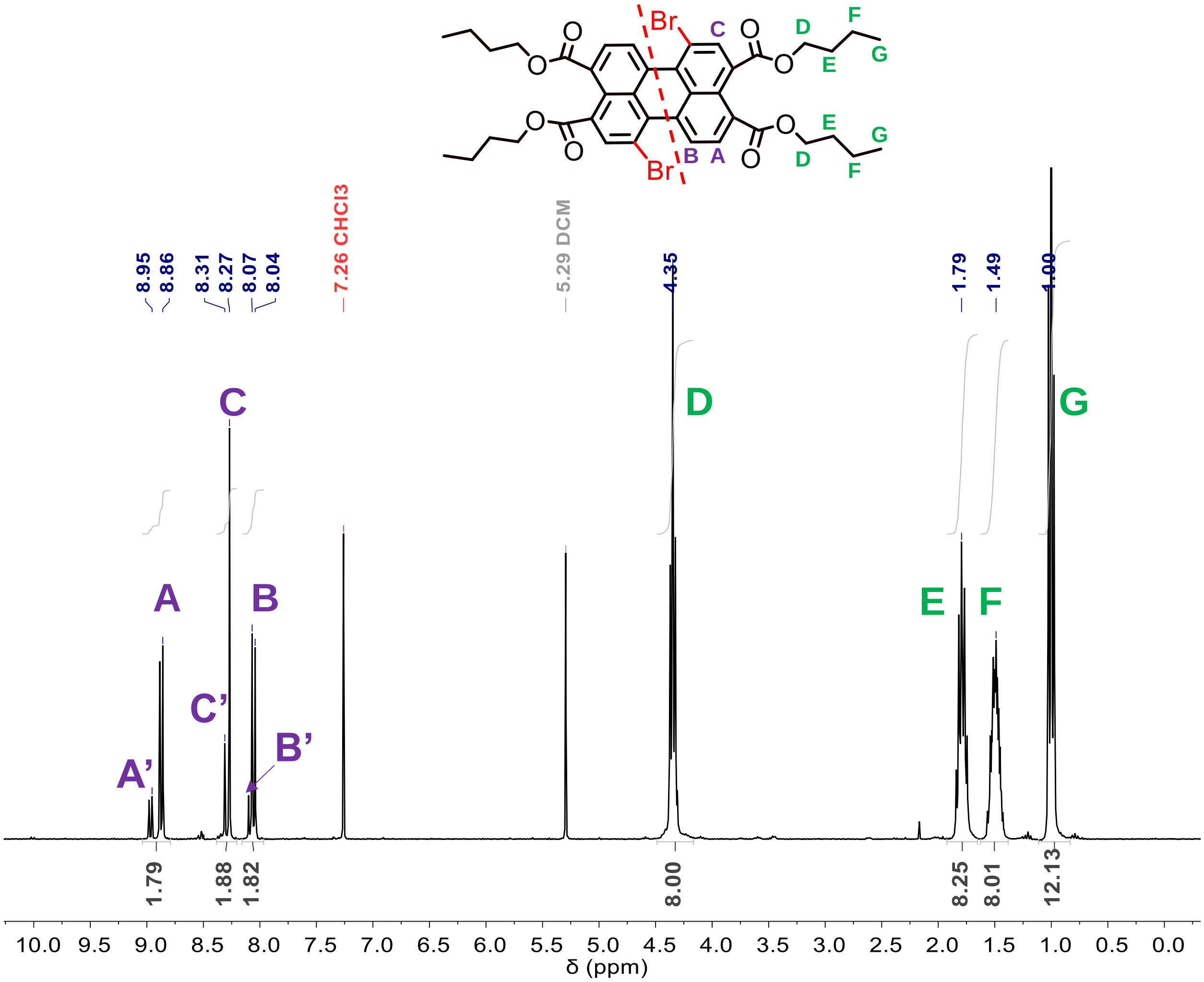

Figure S6: ^{1}H NMR of **(7)** recorded in $CDCl_3$. X' are corresponding to protons from 1,6 bay-substituted isomer[11] which is not separated from the 1,7-bay substituted product.

Synthesis of dibutyl 5,11-dibromo-1,3-dioxo-1H,3H-benzo[10,5]anthra[2,1,9-def]isochromene-8,9-dicarboxylate (8, PMADE Br_2)[8]

PTE Br_2 **(7)** (6.0 g, 7 mmol, 1 eq) and *p*-TsOH.H_2O (2.3 g, 12 mmol, 1.6 eq) were solubilized in 18 mL of *n*-heptane in a single-necked RBF. After 5h of stirring at 90 °C, a red precipitate was formed. The flask was cooled to RT, the solid filtered under vacuum, and washed with water and MeOH. The crude, dark red solid (4.6 g) was used directly for the next step, even though it contains a mixture of symmetrical PDI Br_2, PMADE Br_2 **(8)**, and PTE Br_2 **(7)**. The subsequent step will facilitate the separation of this mixture.

Synthesis dibutyl 5,11-dibromo-2-(2-ethylhexyl)-1,3-dioxo-2,3-dihydro-1H-benzo[10,5]-anthra[2,1,9-def]isoquinoline-8,9-dicarboxylate (9, PMIDE Br_2)

The crude mixture from the previous reaction containing PMADE Br_2 **(8)** (4.6 g, 7 mmol, 1 eq), 2-ethylhexyl amine (1.6 mL, 10 mmol, 1.5 eq) and AcOH (0.2 mL, 3 mmol, 0.5 eq) were solubilized in 115 mL of ethanol in a single-necked RBF. The mixture was stirred overnight under reflux. The product was purified using a silica plug, depositing the crude via dry-loading. Toluene was first used as a solvent to remove the PDI Br_2 as an orange fraction. Then, DCM was used to obtain the target PMIDE Br_2 **(9)** as a yellow fraction (dark red solid, 1.3 g, 22% yield after two steps). The remaining yellow/green fraction corresponds to the PTE Br_2 **(7)**.

δ_H (300 MHz, $CDCl_3$) = 9.21 (H_A, dd, 2H); 8.87 (H_B, s, 1H); 8.65 (H_D, d, 1H); 8.33 (H_C, s, 1H); 8.14 (H_E, d, 1H); 4.36 (H_F, t, 4H); 4.15 (H_G, mult, 2H); 1.95 (H_H, mult, 1H); 1.80 (H_I, quintet, 4H); 1.32-1.51 (H_J, mult, 12H); 1.00 (H_K, t, 6H); 0.95 (H_L, t, 3H); 0.90 (H_L, t, 3H) ppm.

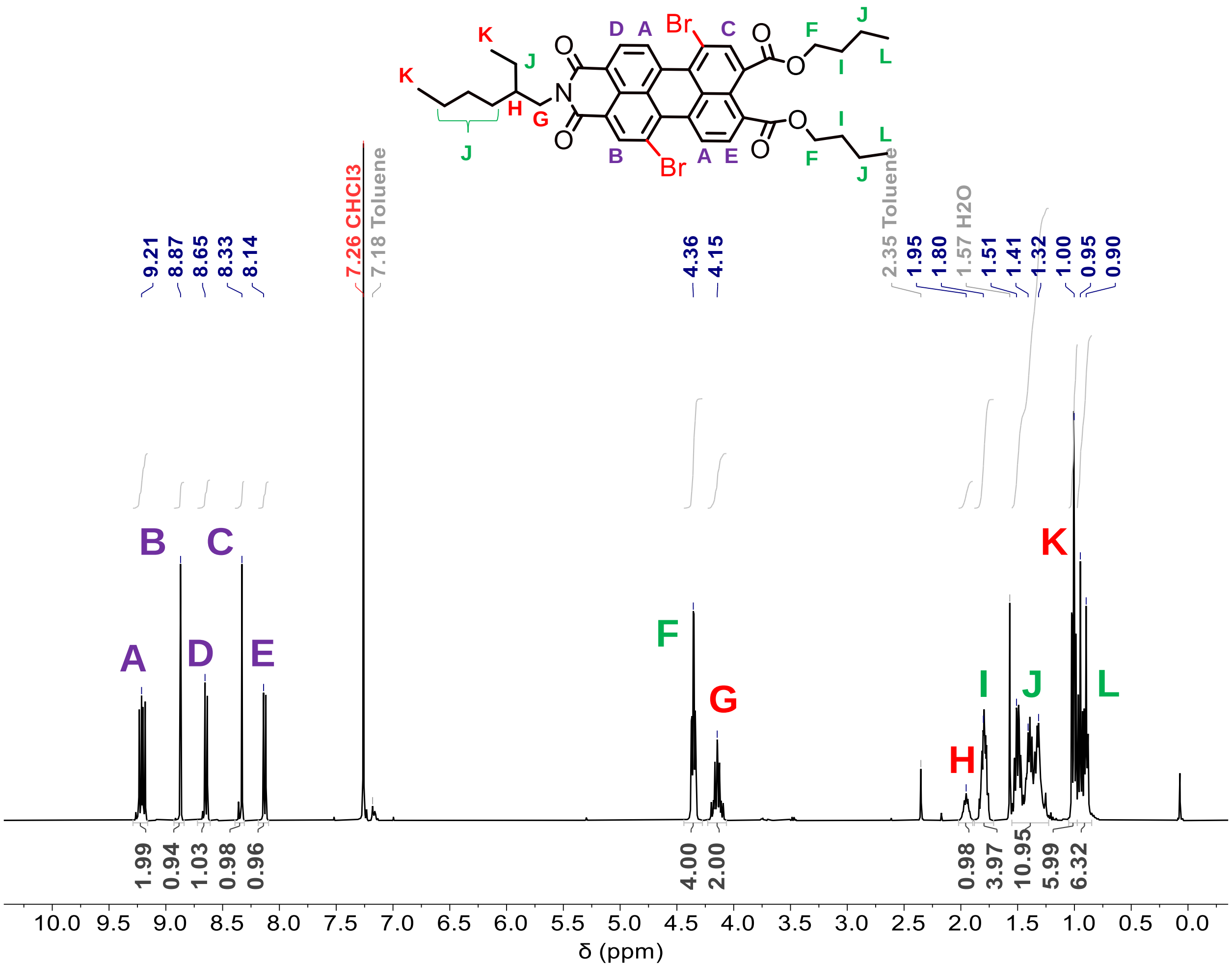

Figure S7: ^{1}H NMR of **(9)** recorded in $CDCl_3$.

Synthesis of 5,12-dibromo-9-(2-ethylhexyl)-1H-isochromeno[6',5',4':10,5,6]anthra[2,1,9-def]isoquinoline-1,3,8,10(9H)-tetraone (10, PMIMA Br_2)[9]

PMIMA Br_2 **(10)** was synthesized using a modified procedure from Sengupta *et al*.[9] PMIDE Br_2 **(9)** (1.3 g, 1.5 mmol, 1 eq), and *p*-TsOH.H_2O (1.96 g, 10 mmol, 6.3 eq) were solubilized in 50 mL of toluene in a single-necked RBF. The mixture was stirred for two days at 90 °C, during which the product formed progressively as a red precipitate. The suspension was cooled to RT, and the solvent was evaporated under reduced pressure. The red solid was dissolved in $CHCl_3$, and the solution was washed three times with distilled water until the pH was neutral. The organic phase was finally dried over $MgSO_4$, filtered, and the solvent removed under reduced pressure. The recovered product (red solid, 1.0 g) was used directly for the next step as it was not soluble enough for ^{1}H NMR analysis.

Synthesis of 5,12-dibromo-2-(2-ethylhexyl)-9-(6-hydroxyhexyl)anthra[2,1,9-def:6,5,10-d'e'f']diisoquinoline-1,3,8,10(2H,9H)-tetraone (11)[10]

PMIMA Br_2 **(10)** (1.0 g, 1.5 mmol, 1 eq), 6-aminohexan-1-ol (0.3 g, 2.5 mmol, 1.7 eq), and AcOH (50 µL, 0.9 mmol, 0.6 eq) were solubilized in 30 mL of ethanol in a single-necked RBF. The mixture was stirred overnight under reflux. The product was purified using a silica plug, depositing the crude via dry loading. DCM was first used as a solvent to remove an orange fraction. Then, a mixture of DCM with 4% of MeOH was used to recover the target non-symmetrical PDI Br_2 **(11)** as an orange fraction. After evaporation of the solvent, a red solid was obtained (0.8 g, 55% yield after two steps).

δ_H (300 MHz, $CDCl_3$) = 9.45 (H_A, d, 2H); 8.90 (H_C, s, 2H); 8.69 (H_B, d, 2H); 4.22 (H_D, mult, 4H); 3.66 (H_E, t, 2H); 1.95 (H_F, mult, 1H); 1.36-1.78 (H_G, mult, 16H); 0.95 (H_H, t, 3H); 0.90 (H_H, t, 3H) ppm.

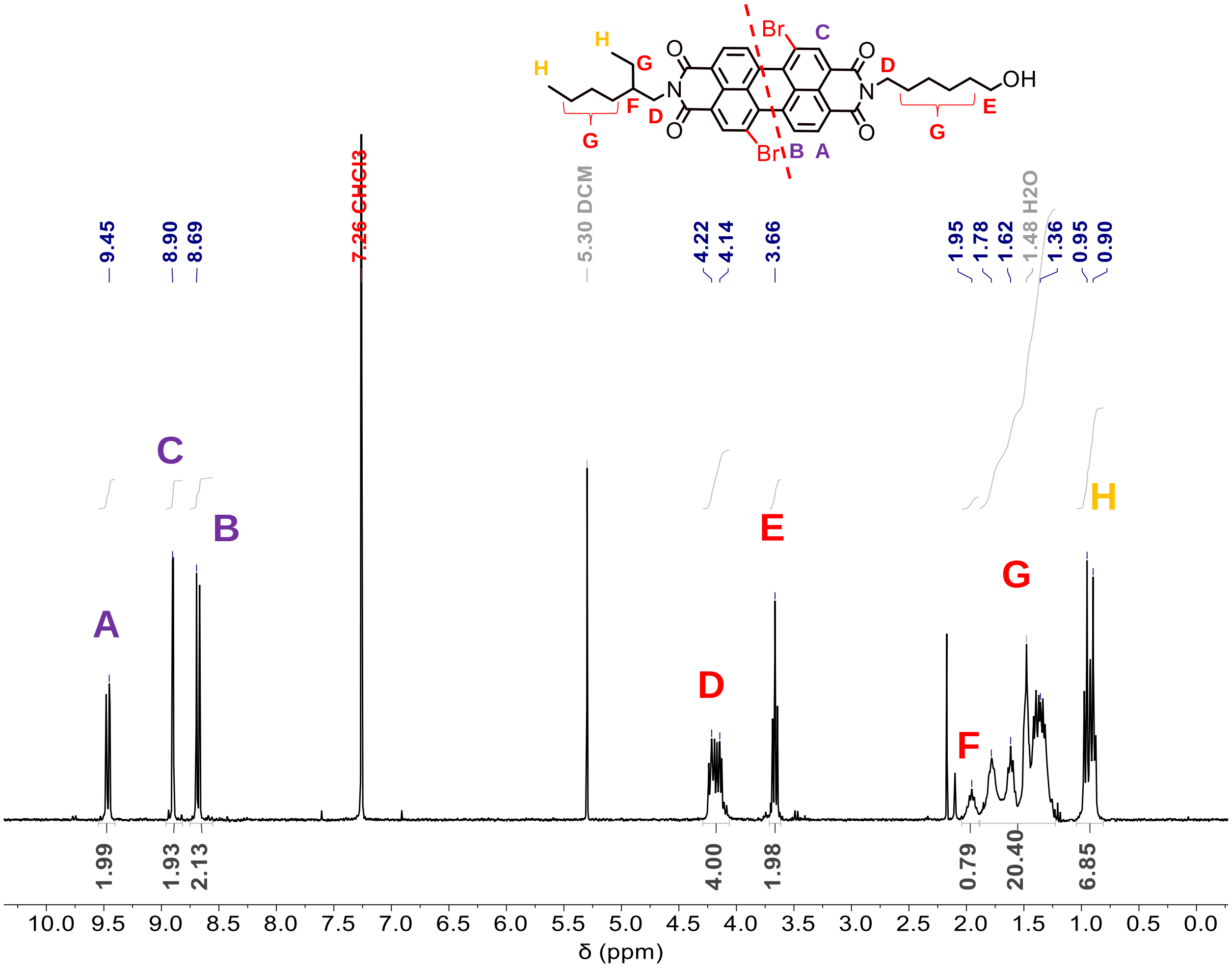

Figure S8: ^1H NMR of **(11)** recorded in $CDCl_3$.

Synthesis of 2-(2-ethylhexyl)-9-(6-hydroxyhexyl)-5,12-bis(2-(2-(2-methoxyethoxy)ethoxy)-ethoxy)anthra[2,1,9-def:6,5,10-d'e'f']diisoquinoline-1,3,8,10(2H,9H)-tetraone (12)

(12) was synthesized using a modified procedure from Mukhopadhyay *et al*.[12] PDI Br_2 **(11)** (791 mg, 1 mmol, 1 eq) was added to a 2-neck RBF (100 mL), set under an argon atmosphere, and solubilized at RT in THF (60 mL, anhydrous). Separately, NaH (161 mg, 6.7 mmol, 6.5 eq) was added in THF (15 mL, dry) under an argon atmosphere at RT, and dry tri(ethylene glycol) monomethyl ether (0.95 mL, 6.5 mmol, 6.2 eq) was added dropwise. After 15 minutes of stirring, the MeTEG solution was added dropwise to the solution of PDI. The initial dark red solution turned dark purple after complete addition and an additional 1h of stirring at RT. The solvent was distilled off and the crude product was solubilized in $CHCl_3$ (150 mL) before washing three times with water to remove the unreacted MeTEG. After drying the purple organic phase over $MgSO_4$, the solvent was removed to give **(12)** as a black solid with a purple tint (498 mg, 52 %).

δ_H (300 MHz, $CDCl_3$) = 9.49 (H_A, d, 2H); 8.48 (H_B, d, 2H); 8.21 (H_C, s, 2H); 4.54 (H_I, t, 4H); 4.17 (H_D, mult, 4H); 4.09 (H_J, t, 4H); 3.55-3.87 (H_{K-N}, mult, 16H); 3.67 (H_E, t, 2H); 3.34 (H_O, s, 6H); 1.94 (H_F, mult, 1H) 1.37-1.79 (H_F, mult, 16H); 0.95 (H_H, t, 3H); 0.90 (H_H, t, 3H) ppm.

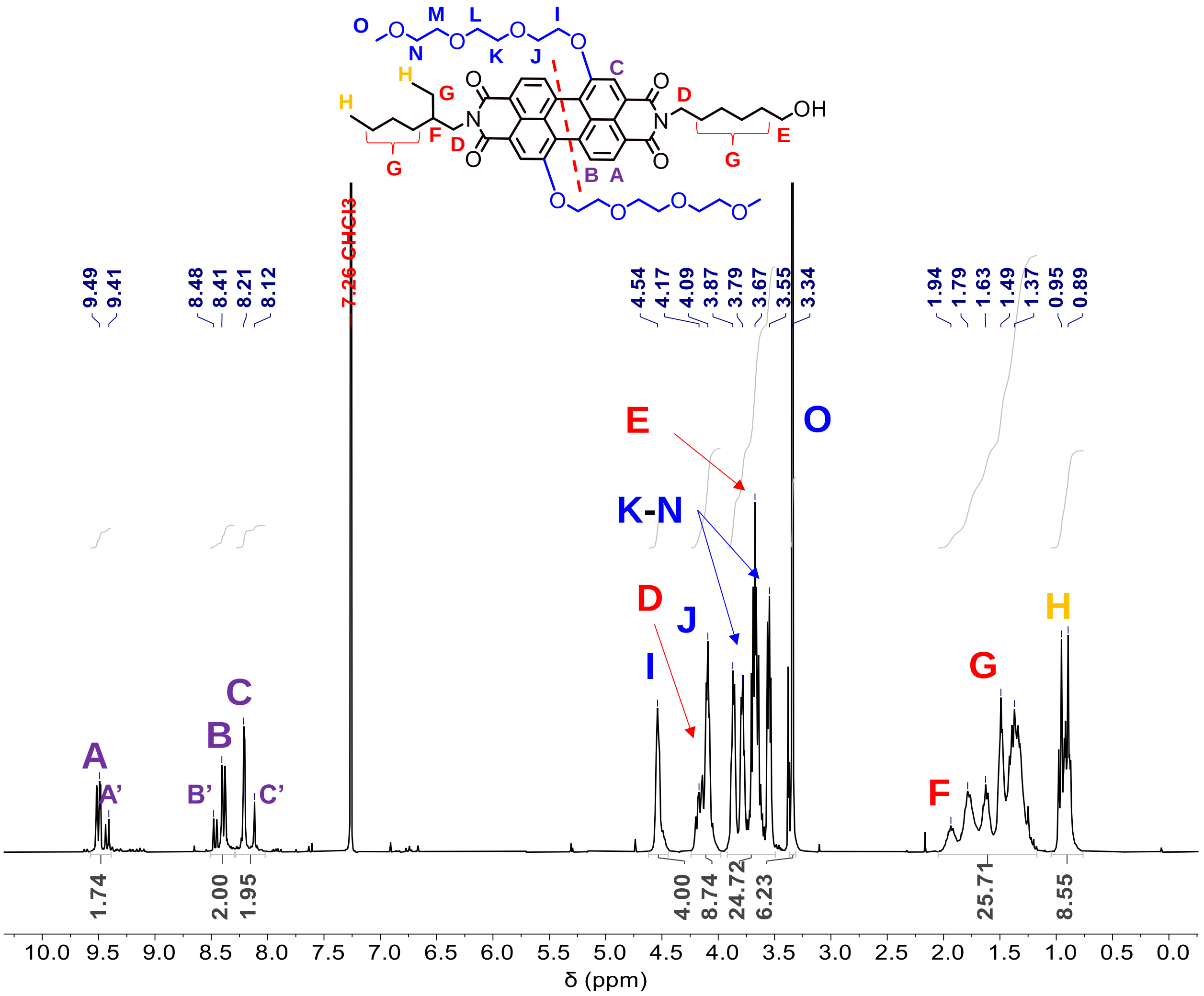

Figure S9: ^{1}H NMR of **(12)** recorded in $CDCl_3$. X' are corresponding to protons from 1,6 bay-substituted isomer[11] which is not separated from the 1,7-bay substituted product.

Synthesis of 6-(9-(2-ethylhexyl)-5,12-bis(2-(2-(2-methoxyethoxy)ethoxy)ethoxy)-1,3,8,10-tetraoxo-3,8,9,10-tetrahydroanthra[2,1,9-def:6,5,10-d'e'f']diisoquinolin-2(1H)-yl)hexyl 3-((((2-cyanopropan-2-yl)thio)carbonothioyl)thio)propanoate (13, PDI-TEG$_2$-TTC)[7]

PDI-TEG$_2$-TTC (13) was synthesized using a procedure from our previous work.[7] **TTC-COOH** (201 mg, 0.9 mmol, 2 eq) was added to a 10 mL Schlenk flask, set under argon before adding $SOCl_2$ (0.3 mL, 4.1 mmol, 10 eq) and stirring the mixture for 10 min at 55 °C to form TTC-COCl (oily liquid). The remaining $SOCl_2$ was removed under vacuum and evaporated using dry toluene (two additions). In parallel, the non-symmetrical PDI MeTEG$_2$ **(12)** (385 mg, 0.4 mmol, 1 eq) was added to a 100 mL RBF and solubilized in 50 mL of dry DCM under argon. Then, the TTC-COCl

was dissolved in 10 mL of dry DCM and added dropwise to the PDI solution at 0 °C. The mixture was stirred for 3 days at 35 °C.

2 mL of EtOH was added to quench the excess TTC-COCl, and the mixture was stirred for 30 min before removing the solvent with a rotary evaporator. The product was precipitated three times in *n*-pentane to remove the free RAFT agent (TTC-COOR). The product was purified using a flash chromatography system, depositing the crude via dry-loading. A mixture of $CHCl_3$ with 0.75% MeOH was used to recover the desired product. The final **PDI-TEG$_2$-TTC (13)** was obtained as a dark purple solid (119 mg, 25%).

δ_H (400 MHz, $CDCl_3$) = 9.54 (H_A, d, 2H); 8.44 (H_B, d, 2H); 8.26 (H_C, s, 2H); 4.56 (H_I, t, 4H); 4.19 (H_D, mult, 4H); 4.10 (H_J, t, 3H); 4.10 (H_E, t, 3H); 3.78-3.87 (H_{K+L}, mult, 8H); 3.59 (H_P, t, 2H); 3.54-3.67 (H_{M+N}, mult, 8H); 3.34 (H_O, s, 6H); 2.75 (H_Q, t, 2H); 1.93 (H_F, mult, 1H); 1.78 (H_R, s, 6H); 1.32-1.68 (H_G, mult, 16H); 0.95 (H_H, t, 3H); 0.89 (H_H, t, 3H) ppm.

δ_C (100 MHz, $CDCl_3$) = 217 ($C_{C=S}$), 171 ($C_{C=O\ ester}$), 163 ($C_{C=O\ imide}$), 120-156 ($12C_{aromatic}$), 117 ($C_{nitrile}$), 65-72 ($7C_{MeTEG}$), 59 ($C_{CH2\text{-}O}$), 10-44 ($17C_{alkyl}$) ppm.

HRMS (ESI) m/z: [M+Na]$^+$ Calculated: ($C_{46}H_{47}N_3O_6S_3Na$); 1180.43 **Found:** 1180.4296

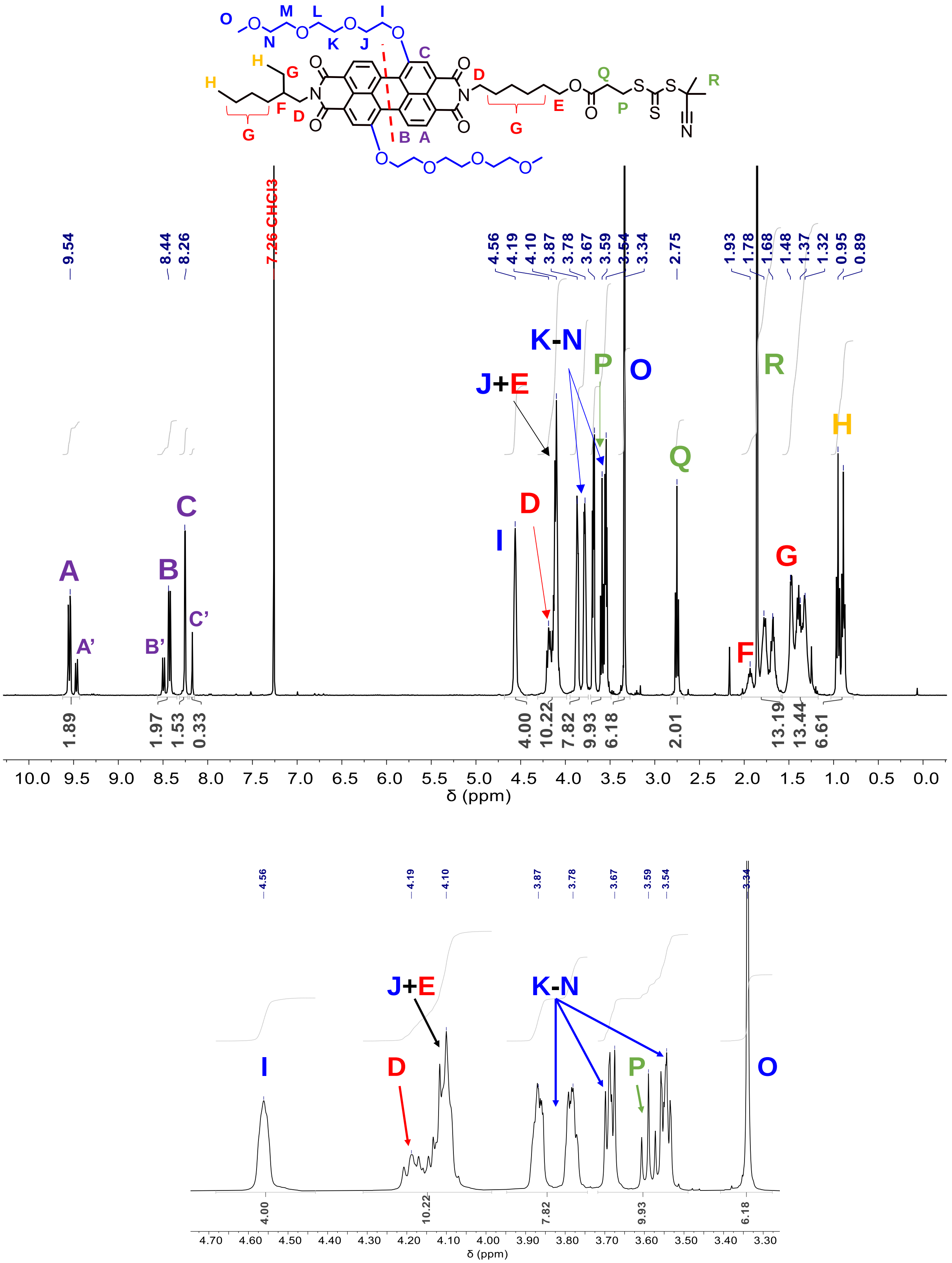

<u>Figure S10:</u> ^{1}H NMR of the final **PDI-TEG$_2$-TTC (13)** recorded in $CDCl_3$. X' are corresponding to protons from 1,6 bay-substituted isomer[11] which is not separated from the 1,7-bay substituted product. Spectrum zoomed between 3.3 and 4.7 ppm.

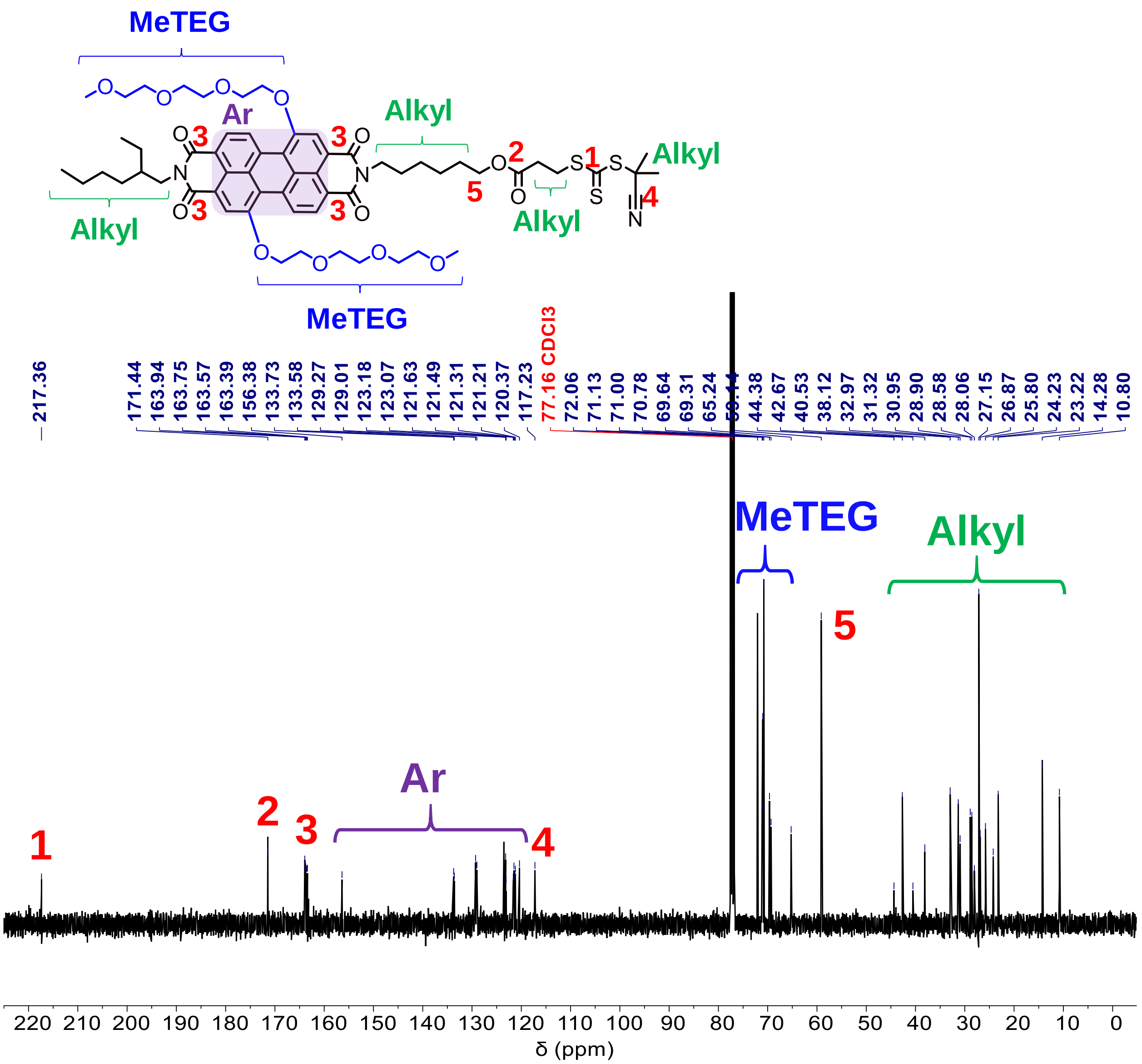

<u>Figure S11:</u> ^{13}C NMR of the final **PDI-TEG$_2$-TTC (13)** recorded in $CDCl_3$.

Additional characterizations for the PDI RAFT agents

ε determination in THF (calibration curve)

The final **PDI-TTC (6)** and **PDI-TEG$_2$-TTC (13)** were analyzed by UV/Vis spectroscopy in THF **(Figure S12a, d)**.

Solutions at different concentrations (ranging from 5.10^{-7} mol/L to 10^{-5} mol/L) were prepared from the dilution of a stock solution at ca. 3.10^{-4} mol/L. To prepare these solutions, 14.3 mg of **PDI-TTC (6)** and 18.3 mg of **PDI-TEG$_2$-TTC (13)** were separately solubilized in THF in a 20 mL volumetric flask.

The molar extinction coefficients (ε) of **PDI-TTC (6)** and **PDI-TEG$_2$-TTC (13)** in THF were determined at 522 and 563 nm, respectively, using the Beer-Lambert law (calibration curve A = ε.l.C, where A is the absorbance measured, l = 1 cm the length of the cuvette and C the molar concentration – valid for A < 1) **(Figure S12b, e)**.

Characterization by SEC (diluted conditions, C = 0.1 mg/mL) in DMF (+ LiBr 1 g/L) was also performed **(Figure S12c, f)** to determine their elution volume, which can be used for comparison with the polymers synthesized.

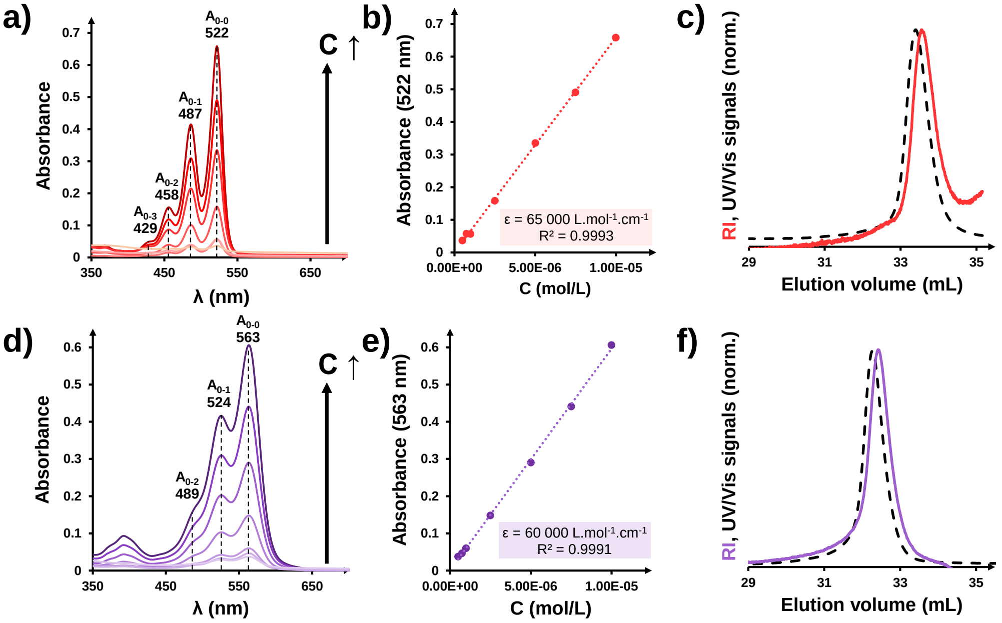

Figure S12: a) UV/Vis absorption spectra of **PDI-TTC (6)** in THF at different concentrations from 5.10^{-7} to 10^{-5} mol/L, recorded at 20 °C, b) Absorbance versus concentration plot, Beer Lambert law at 520 nm to determine the extinction coefficient of **PDI-TTC (6)** in THF, ε = 65 000 L.mol^{-1}.cm^{-1}. c) Normalized SEC traces of **PDI-TTC (6)** in DMF (+ LiBr) with RI (red, solid line) and UV/Vis (black, dashed line) signals. The UV/Vis detector was set at 520 nm. d) UV/Vis absorption spectra of **PDI-TEG$_2$-TTC (13)** in THF at different concentrations from 5.10^{-7} to 10^{-5} mol/L, recorded at 20 °C, e) Absorbance versus concentration plot, Beer Lambert law at 563 nm to determine the extinction coefficient of

PDI-TEG$_2$-TTC (13) in THF, ε = 60 000 L.mol^{-1}.cm^{-1}. f) Normalized SEC traces of **PDI-TEG$_2$-TTC (13)** in DMF (+ LiBr) with RI (purple, solid line) and UV/Vis (black, dashed line) signals. The UV/Vis detector was set at 560 nm.

DFT calculations of the dihedral angles

The structures of the PDI RAFT agents were simplified for the structure optimization, with only CH_3 added at the *N*-imide position since only bay-functionalization has a measurable impact on the twisting of the aromatic core.[11] Also, only ethylene glycol (EG) unit (instead of three repeating units) was added to the bay-positions, given that the core twisting (loss of planarity) is mainly influenced by the nature of the atom directly linked to the PDI core.[11]

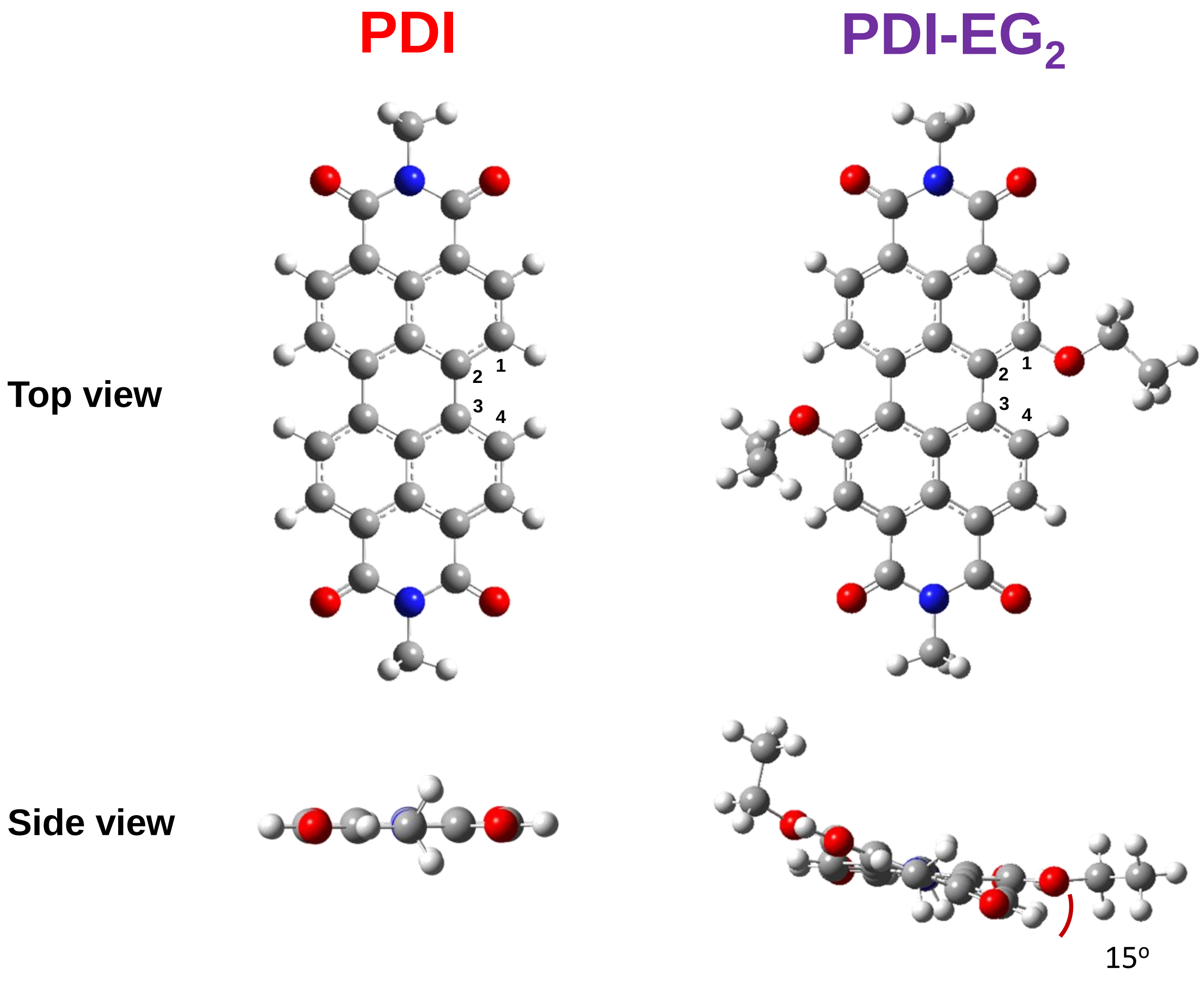

Figure S13: DFT calculations on simplified structures of **PDI-TTC** (PDI) and **PDI-TEG$_2$-TTC** (PDI-EG$_2$). The dihedral angle displayed is corresponding to the angles between the carbons 1,2,3 and 4.

b) Synthesis of PDI-functional PDMAc[13]

The same synthetic procedure was used for both types of polymers (using **PDI-TTC (6)** or **PDI-TEG$_2$-TTC (13)** as RAFT agent).

In a typical experiment (entry **P3-TEG**, **Table S1**), a 1.5 mL screw-cap septum glass vial was loaded with 9.9 mg (8.55 μmol) of **PDI-TEG$_2$-TTC (13)**, 0.1762 g (1.78 mmol) of DMAc, 30 mg (0.32 mL) of DMF, 0.0863 g of an AIBN solution in 1,4-dioxane (9.8 mg of AIBN dissolved in 4.9880 g of 1,4-dioxane, resulting in adding 1.03 μmol of AIBN) and 0.6150 g of 1,4-dioxane. The mixture was degassed under argon in a water bath at 10 °C for 30 min. The flask was then immersed in an oil bath at 70 °C. Aliquots were regularly taken from the reaction medium and analyzed by ^{1}H NMR in $CDCl_3$ to determine the conversion in DMAc, using the three vinylic protons of the monomer (signals between δ = 5.5 and 6.5 ppm) and DMF as an internal reference (δ = 7.9 ppm). After 21h of reaction, the polymerization was stopped by immersing the flask in an ice bath and opening it to air.

For the PDI-PDMAc (TEG-free) syntheses, the polymer was passed through neutral alumina, using THF as eluent to remove remaining monomer and PDI impurities, then a mixture of $CHCl_3$ with 4% of MeOH was used to recover the polymer. The procedure was repeated three times. For the PDI-TEG$_2$-PDMAc syntheses, the polymer was precipitated three times in cold Et_2O to remove any remaining monomer. After the last purification, the purple powder was solubilized in DCM, followed by solvent evaporation and drying under vacuum.

The final polymers were characterized by SEC in DMF (+ LiBr 1 g/L) and ^{1}H NMR in $CDCl_3$ **(Figure S14 and Figure S15)**.

Table S1: Polymerization conditions and structural characterization of PDI-functional PDMAc.

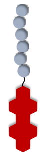
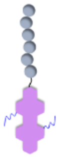

Entry	M, I, Solvent, T	$[M]_0$ [a] *(mol/L)*	$[M]_0/[TTC]_0$ [b]	$[TTC]_0/[I]_0$ [c]	Conv. [d] (%)	$DP_{n,\,conv.}$ [d]	$DP_{n,\,NMR}$ [e]	$M_{n,\,NMR}$ [e] *(kg/mol)*	$M_{n,\,SEC}$ [f] *(kg/mol)*	*Đ* [f]
P1	DMAc, AIBN, 1,4-dioxane, 70 °C	2.9	138	9	5	7	17	2.5	1.9	1.20
P2	DMAc, AIBN, 1,4-dioxane, 70 °C	2.8	135	10	11	15	24	3.2	2.6	1.22
P3	DMAc, AIBN, 1,4-dioxane, 70 °C	2.8	492	9	10	51	134	14.1	8.8	1.42
P1-TEG	DMAc, AIBN, 1,4-dioxane, 70 °C	2.7	171	9	7	12	15	2.6	2.4	1.20
P2-TEG	DMAc, AIBN, 1,4-dioxane, 70 °C	2.7	132	10	10	16	24	3.6	3.4	1.19
P3-TEG	DMAc, AIBN, 1,4-dioxane, 70 °C	2	208	8	37	77	120	13.1	8.6	1.16

a) Initial molar concentration of DMAc (=M) introduced,
b) Initial molar ratio of DMAc/PDI RAFT agent introduced,
c) Initial molar ratio of PDI RAFT agent/initiator introduced,
d) Monomer conversion and number-average degree of polymerization DP_n at the end of the polymerization,
e) Number-average degree of polymerization, DP_n, and number-average molar mass, M_n, determined by ^1H NMR in $CDCl_3$ on the precipitated polymer,
f) Number-average molar mass, M_n, and dispersity, *Đ*, determined by SEC in DMF (+ LiBr, 1 g/L) on the precipitated polymer using a PMMA calibration.

Characterization by SEC

The M_n and $Đ$ of the different polymers synthesized were determined by SEC **(Figure S14)**. For **P1** and **P2**, the molar percentage of remaining RAFT agent was obtained by deconvolution of the UV/Vis chromatogram.

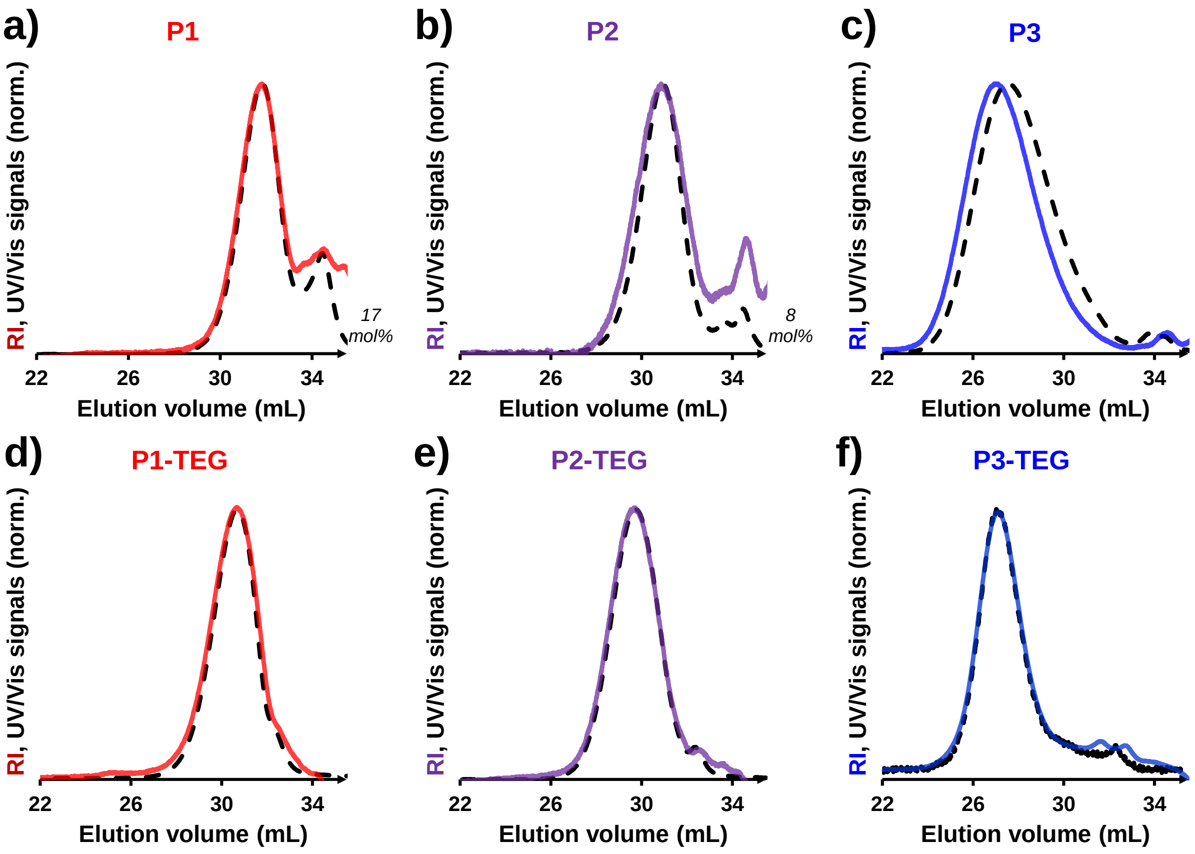

Figure S14: Overlay of the normalized SEC RI (solid lines) and UV/Vis (at 520 nm for PDI-PDMAc (TEG-free) and at 560 nm for PDI-TEG$_2$-PDMAc) (dashed lines) traces of the purified polymers a) **P1**, b) **P2**, c) **P3**, d) **P1-TEG**, e) **P2-TEG**, f) **P3-TEG** in DMF (+ LiBr 1g/L). For **P1** and **P2** the molar percentage of remaining RAFT agent is indicated on the right of the chromatogram.

Characterization by ^{1}H NMR

^{1}H NMR was used to determine the DP_n of the different polymers **(Figure S15)**. For the PDI-PDMAc (TEG-free) polymers, the integration of protons B (2*CH_2 imide and 1*CH_2 next to the ester) was set at 6H, and for the PDI-TEG$_2$-PDMAc, the integration of the aromatic protons A at 9.7 ppm was set at 2H. These integrations were compared to the integration of characteristic protons of the polymer (protons a and b, between 2.5 and 3.2 ppm). From this integration, the

integration of protons O must be removed. For both **P1** and **P2**, the molar percentage of impurities (determined by GPC) was also considered to calculate the $DP_{n,\ NMR}$.

$DP_{n,\ NMR}$ was then calculated according to **Equation 1**.

$$DP_{n\ NMR} = \frac{\frac{\mathrm{Int_a} + \mathrm{Int_b} - \mathrm{Int_O}}{7}}{\frac{\mathrm{Int_A}}{2}}$$

Equation 1: Calculation of the DP_n by ^{1}H NMR.

a)

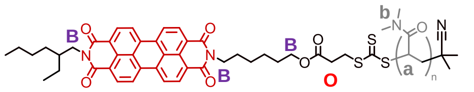

$$DP_{n\,\mathbf{P1}} = \frac{104.77 + (104.77\text{x}0.17) - 2}{7} = 17.2$$

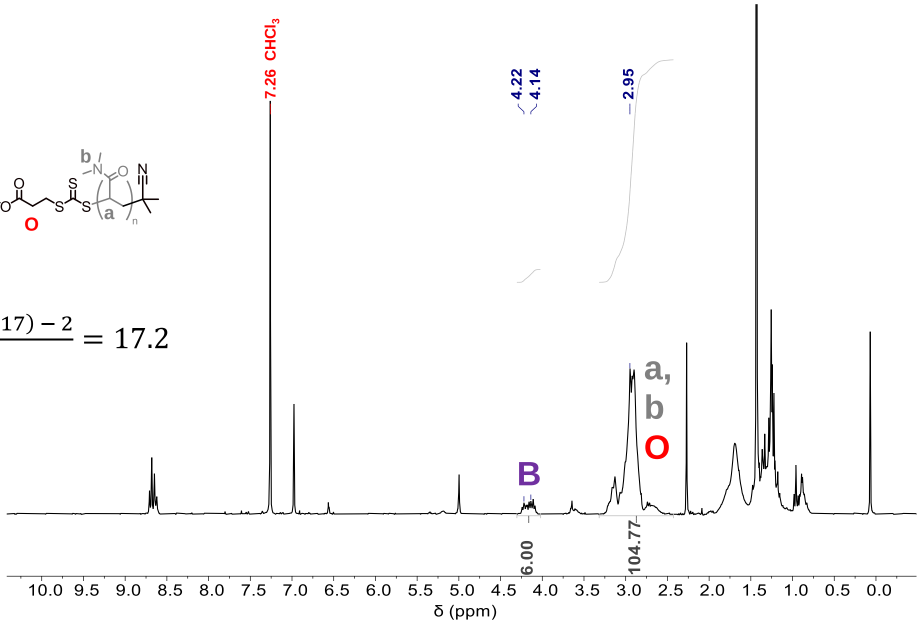

b)

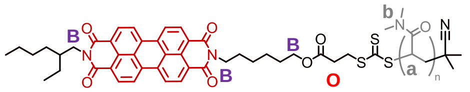

$$DP_{n\,\mathbf{P2}} = \frac{156.25 + (156.25\text{x}0.08) - 2}{7} = 23.8$$

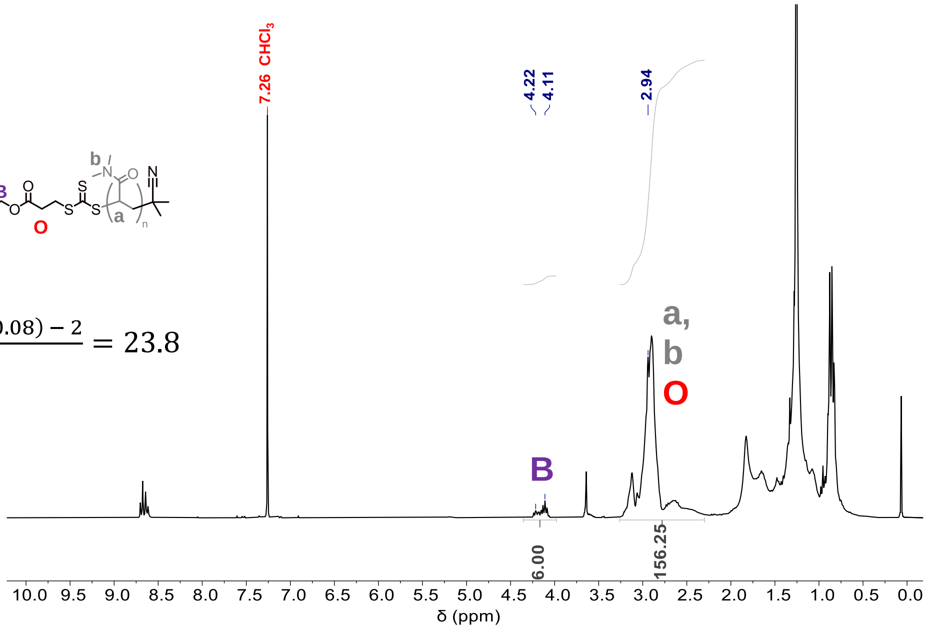

c)

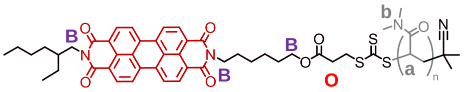

$$DP_{n\,\mathbf{P3}} = \frac{938.99 - 2}{7} = 133.9$$

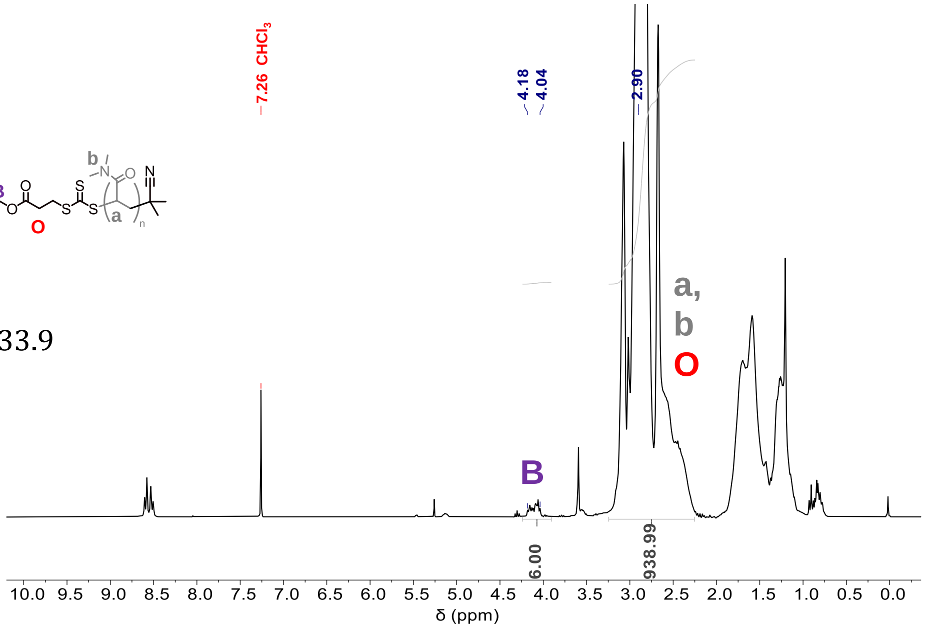

d)

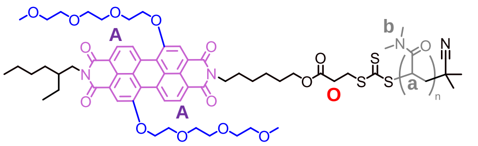

$$DP_{n\,\mathbf{P1\text{-}TEG}} = \frac{108.64 - 2}{7} = 15.2$$

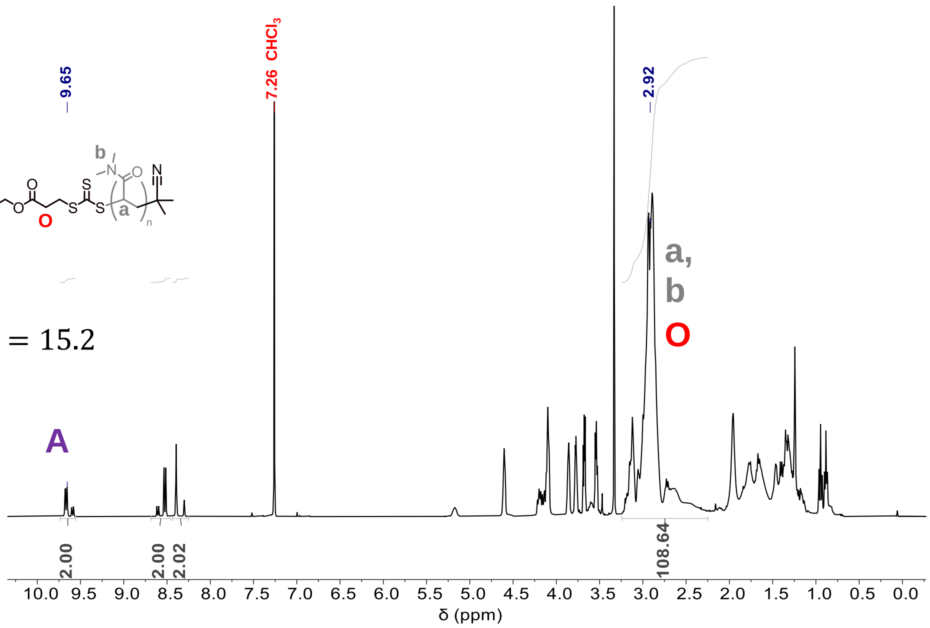

e)

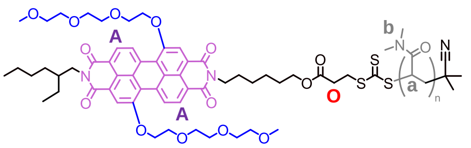

$$DP_{n\,\mathbf{P2\text{–}TEG}} = \frac{171.88 - 2}{7} = 24.3$$

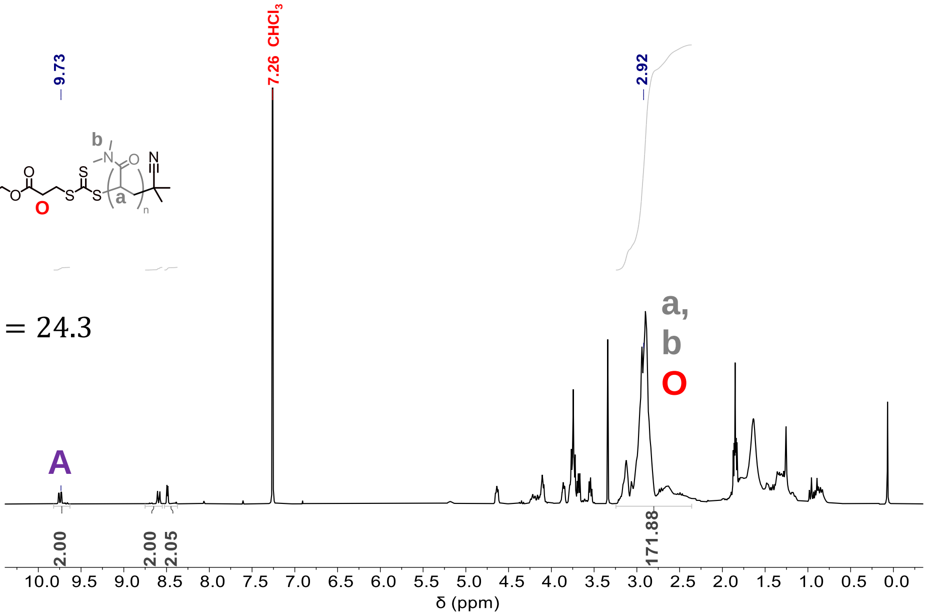

f)

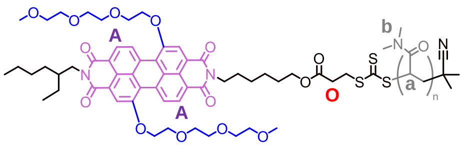

$$DP_{n\,\mathbf{P3\text{–}TEG}} = \frac{842.59 - 2}{7} = 120.1$$

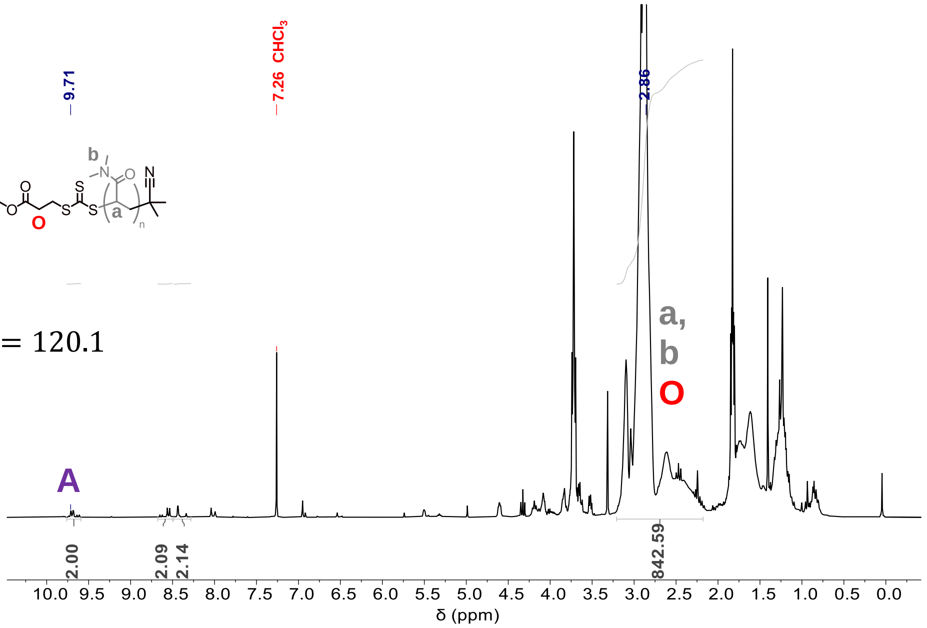

<u>Figure S15:</u> ^{1}H NMR spectra of the purified polymers a) **P1**, b) **P2**, c) **P3**, d) **P1-TEG**, e) **P2-TEG** and f) **P3-TEG** in $CDCl_3$.

4. Self-assembly in water

a) Direct dissolution in water.

As described above, whatever the purification process (alumina plug or precipitation), the final polymers were dissolved in DCM, and this solution was evaporated under reduced pressure to obtain the solid polymer. The solid was directly dissolved in H_2O to reach a final concentration of 10 g/L. **P2** and **P3** PDI-PDMAc (TEG free) dissolved within 5 min, while **P1** dissolved only after stirring overnight at RT. For **P1**, a small amount of precipitate appeared over time and was redispersed after shaking. Visually, all PDI-TEG$_2$-PDMAc dissolved completely within 5 min, except for the **P1-TEG,** which fully dissolved after 15 min. The solutions were then stored at 5 °C.

Cryo-TEM analyses were performed at 10 g/L in H_2O, a few days after dissolution (between 1 and 11 days).

SAXS analyses were performed at 10 g/L in H_2O. SAXS data were fitted with models using SasView. **P1**, **P1-TEG**, and **P2-TEG** were fitted with a cylinder model, and **P3** and **P3-TEG** were fitted with a sphere model. **P2** was fitted with a plugin model combining spheres and cylinders, as the fits with sphere and cylinder models alone were not sufficient to fit the data over the whole q range **(Figure S17)**. All fitting details are displayed in **Tables 1 & 2** in the main article.

UV/Vis analyses were performed at different times after dissolution in H_2O by taking aliquots of the 10 g/L solution and diluting it to 0.1 g/L before analyses.

Fluorescence spectra in THF were performed at 0.02 g/L to obtain Abs $\lesssim$ 0.1 at the excitation wavelength. For **P2**, the excitation wavelength was at 490 nm, and for **P2-TEG**, it was set at 520 nm.

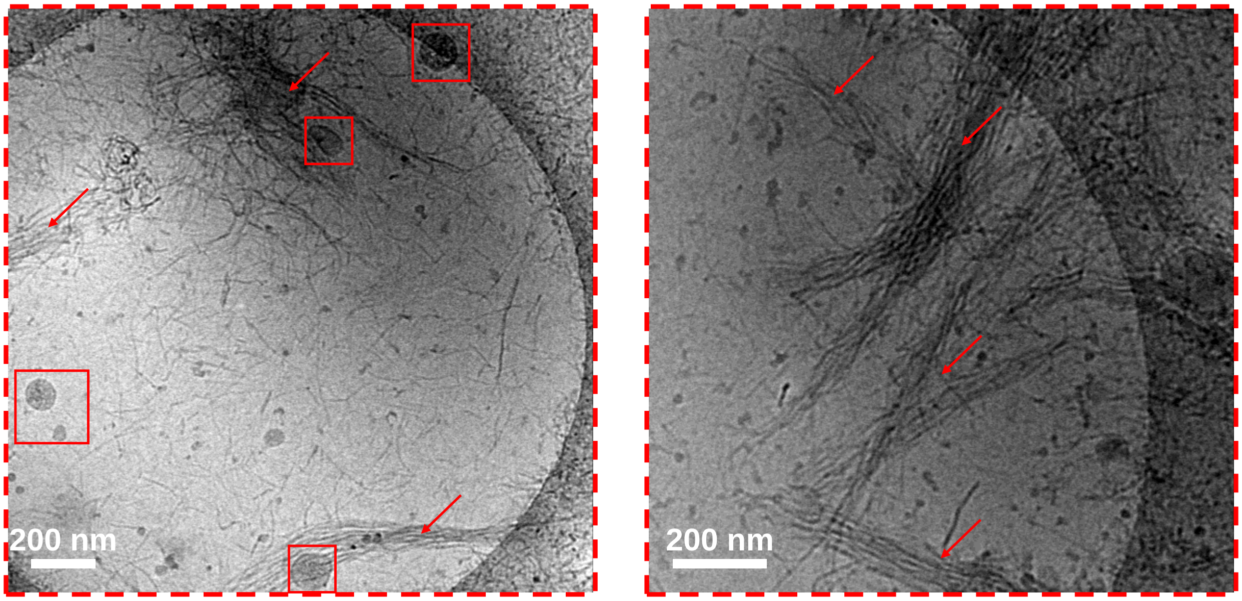

Figure S16: Representative cryo-TEM pictures of the 10 g/L aqueous solution of **P1**, showing the presence of a majority of bundles of cylinders (arrows) and a few vesicles (squares). The dark spots are surface contaminations stemming from water crystals.

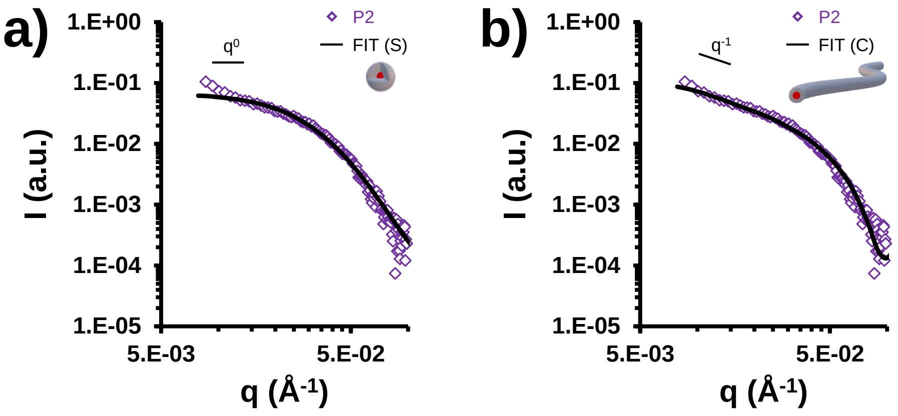

c)

**Entry**	**Morpho. SAXS**	**Dim. SAXS** *(nm)* **(σ)**
**P2**	Spheres	D (S) = 6.8 (0.39)
	Cylinders	D (C) = 8 (0.2)<br>L (C) = 45 (0)

Figure S17: SAXS traces of **P2** at 10 g/L in H_2O fitted with a) sphere (S) and b) cylinder (C) models. The black lines are models fitting the data. These fits highlight the necessity to use a combination of a sphere model and a cylinder model to fit the data. c) Geometrical parameters of the self-assemblies obtained by SAXS. Dimensions of the nano-objects determined by fitting SAXS data, with lognormal polydispersity in parentheses. (0) means that no polydispersity was considered in the fitting.

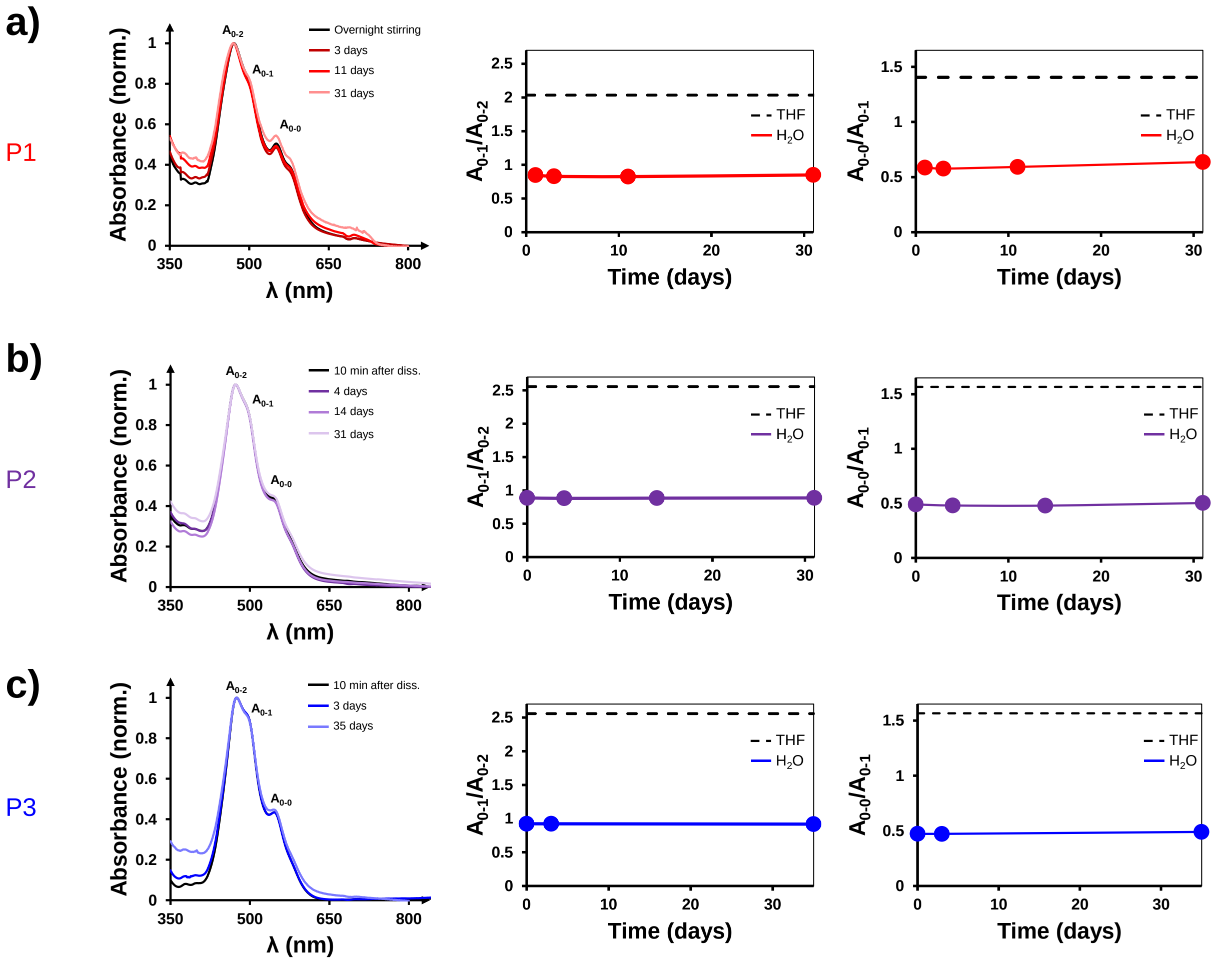

Figure S18: (left) Normalized UV/Vis absorbance spectra at 0.1 g/L in water, with corresponding (middle) $A_{0\text{-}1/0\text{-}2}$ and (right) $A_{0\text{-}0}/A_{0\text{-}1}$ ratio in THF (black, dashed lines) and H_2O (colored, circles with solid lines) of a) **P1**, b) **P2**, c) **P3** at different times after dissolution in H_2O.

Fluorescence spectroscopy also proved to be a useful tool to study these polymer systems, given its sensitivity and high resolution. As observed with the absorbance, the bay-substitution of the PDI also leads to a red shift of the emission maximum in THF **(Figure S19)**. Larger Stokes shifts were also observed with the TEG-functionalized PDIs (26 nm) in comparison to the TEG-free PDI polymers (9 nm).

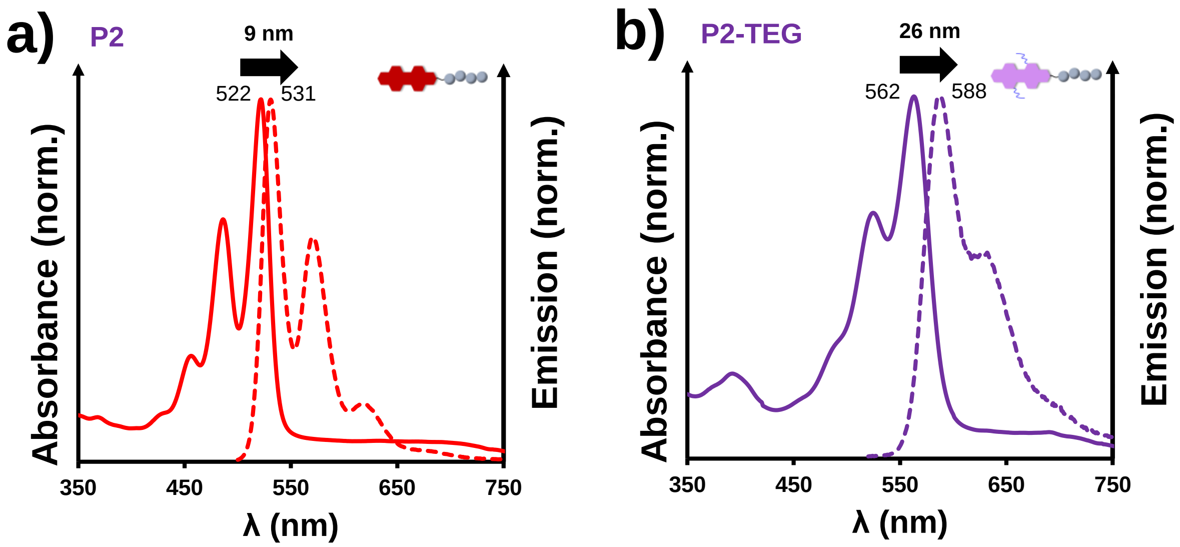

<u>Figure S19:</u> Normalized UV/Vis absorbance (solid lines) and Fluorescence (dashed lines) emission spectra of a) **P2** (λ_{ex} = 490 nm) and b) **P2-TEG** (λ_{ex} = 520 nm) at 0.02 g/L in THF. Stokes shifts are 1360 cm^{-1} and 787 cm^{-1} for **P2** and **P2-TEG**, respectively.

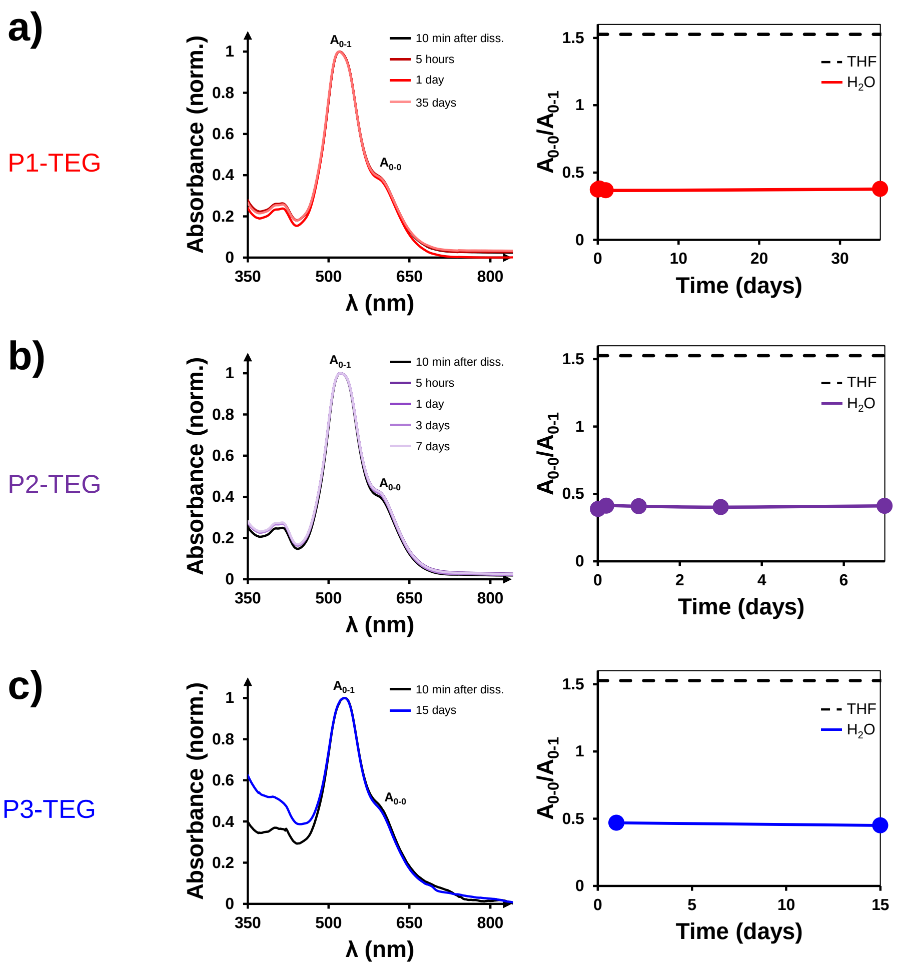

Figure S20: (left) Normalized UV/Vis absorbance spectra at 0.1 g/L in water, with corresponding (right) $A_{0\text{-}0}/A_{0\text{-}1}$ ratio in THF (black, dashed lines) and H_2O (colored, circles with solid lines) a) **P1-TEG**, b) **P2-TEG**, c) **P3-TEG** at different times after dissolution in H_2O.

b) Effect of temperature (and time).

The freshly prepared 10 g/L aqueous polymer solutions were heated for 10 min at 60 °C using an oil bath and analyzed directly after by cryo-TEM. Visually, **P1-TEG** and **P2-TEG** remained transparent, while **P1-TEG** became more viscous. The **P1-TEG** solution at 10 g/L in H_2O was aged for 10 months. All polymer solutions were stored at 5 °C.

Viscometry experiments on **P1-TEG** were performed at 3 g/L by diluting the (freshly prepared) 10 g/L solution with H_2O.

Table S2: Morphologies and corresponding mean diameter and length values with standard deviations determined from cryo-TEM images of **P1-TEG** (with at least 30 measurements), freshly prepared and aged 10 months after dissolution in H_2O, after heating the freshly prepared solution 10 min at 60°C, and aged 2 months after heating (cryo-TEM images are displayed in **Figure 3** in the main article). We cannot exclude that these length values are underestimated because only cylinders shorter than 2.1 µm can be treated using ImageJ from the cryo-TEM images.

Entry	Morpho. cryo-TEM	$D_{cryo\text{-}TEM}$ *(nm)*	$L_{cryo\text{-}TEM}$ *(nm)*
**Freshly prepared, RT**	Cylinders	7.8 ± 1.1	190 ± 151
**Aged 10 months, RT**	Long Cylinders	7.7 ± 0.9	1033 ± 544
**Freshly prepared, 60°C, 10 min**	Long Cylinders	7.3 ± 1.0	1152 ± 498
**Aged, 2 months after heating**	Long Cylinders	7.0 ± 0.9	1058 ± 400

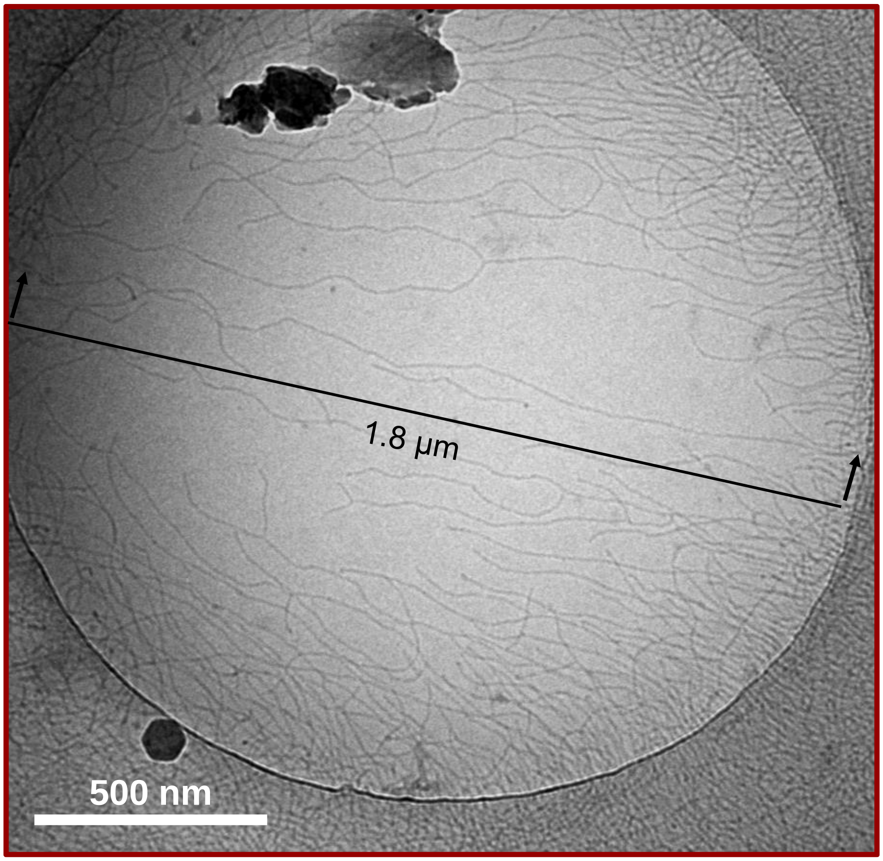

Figure S21: Representative cryo-TEM pictures of **P1-TEG** after heating for 10 min at 60 °C the freshly prepared 10 g/L solution, showing the formation of long nanocylinders up to micrometer length. The dark spots are surface contaminations stemming from water crystals.

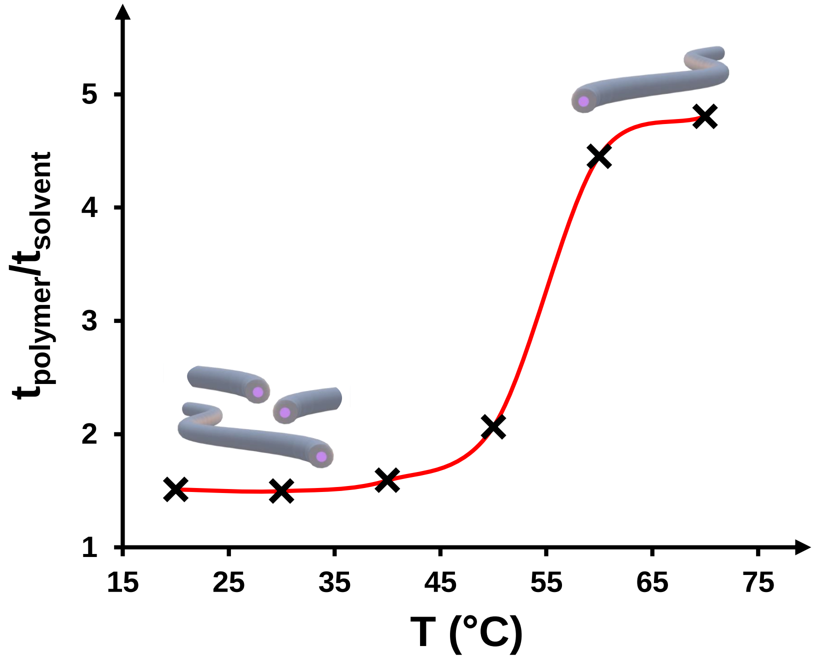

Figure S22: Relative viscosity of a **P1-TEG** solution at 3 g/L in water, as a function of temperature. The large increase in viscosity above 50°C demonstrates the formation of long cylinders after heating.

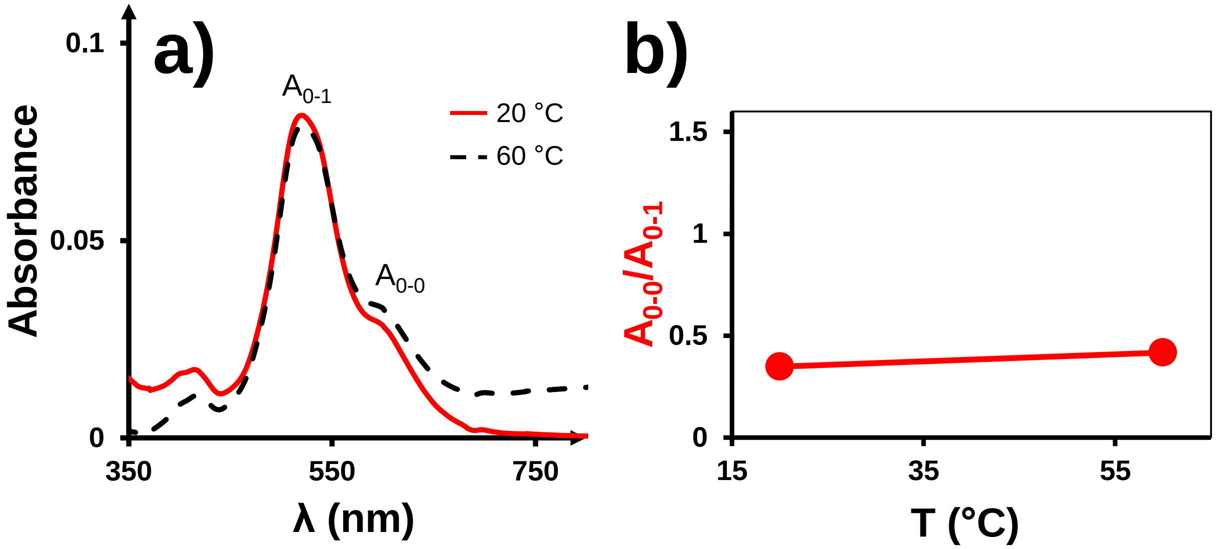

Figure S23: a) UV/Vis absorbance spectra in water of **P1-TEG** at 20 °C (red, solid lines) and 60 °C (black, dashed lines) with b) corresponding A_{0-0}/A_{0-1} ratio as a function of temperature.

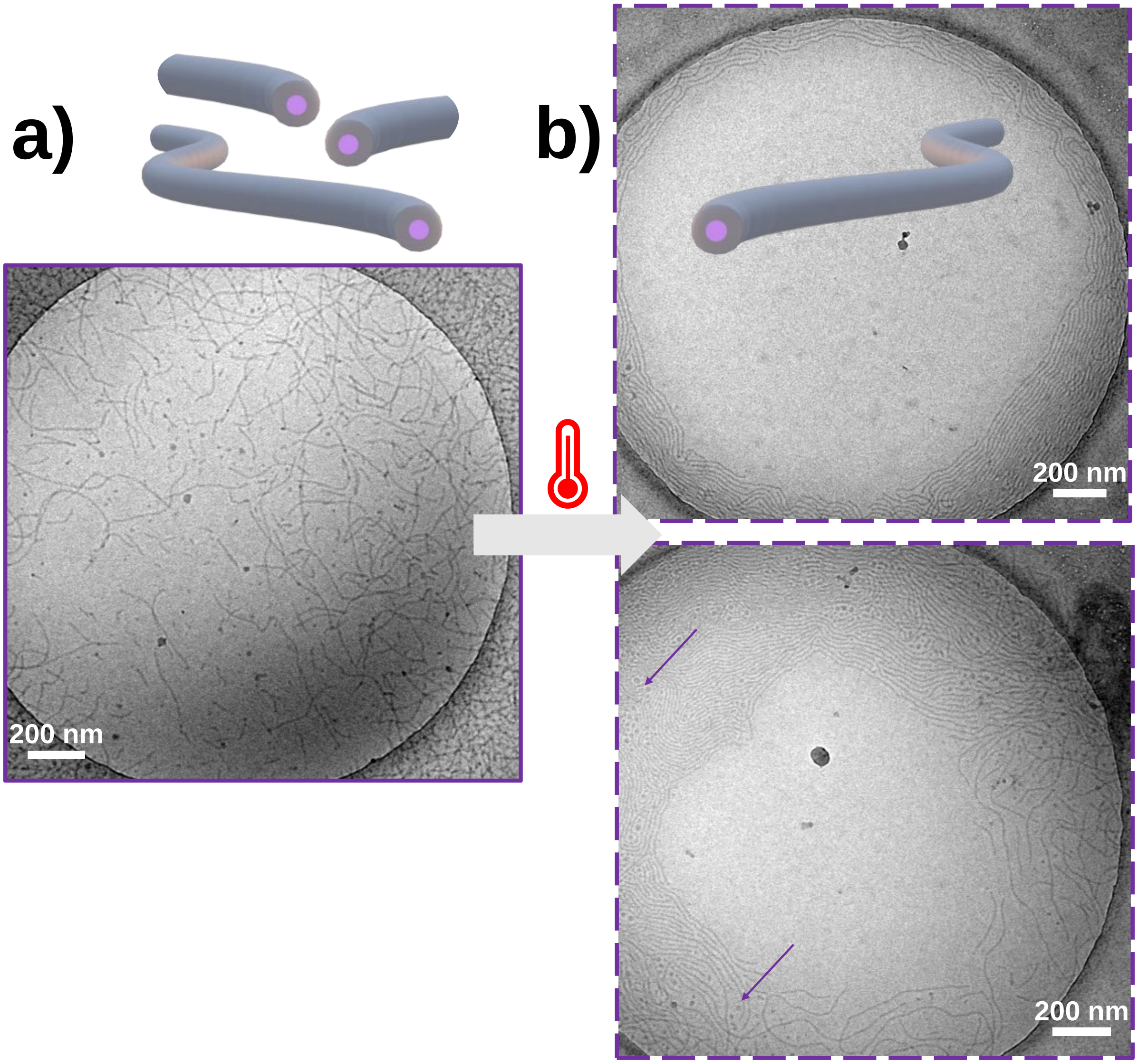

Figure S24: Representative cryo-TEM pictures of the 10 g/L aqueous solution of **P2-TEG** a) at 20 °C and b) after heating 10 min at 60 °C. The arrows indicate representative spherical aggregates. The dark spots are surface contaminations stemming from water crystals.

Table S3: Morphologies and corresponding mean diameter and length values with standard deviations determined from cryo-TEM images of **P2-TEG** (with at least 30 measurements), of the 10 g/L solutions, not heated and heated 10 min at 60°C (cryo-TEM images are displayed in **Figure S23**). We cannot exclude that these lengths values are underestimated because only cylinders shorter than 2.1 µm can be treated using ImageJ from the cryo-TEM images.

Entry	Morpho. cryo-TEM	$D_{cryo\text{-}TEM}$ *(nm)*	$L_{cryo\text{-}TEM}$ *(nm)*
**Freshly prepared, RT**	Cylinders	8.1 ± 1.3	159 ± 123
**Freshly prepared, 60°C, 10 min**	Long Cylinders (+spheres)	8.0 ± 1.1	829 ± 384

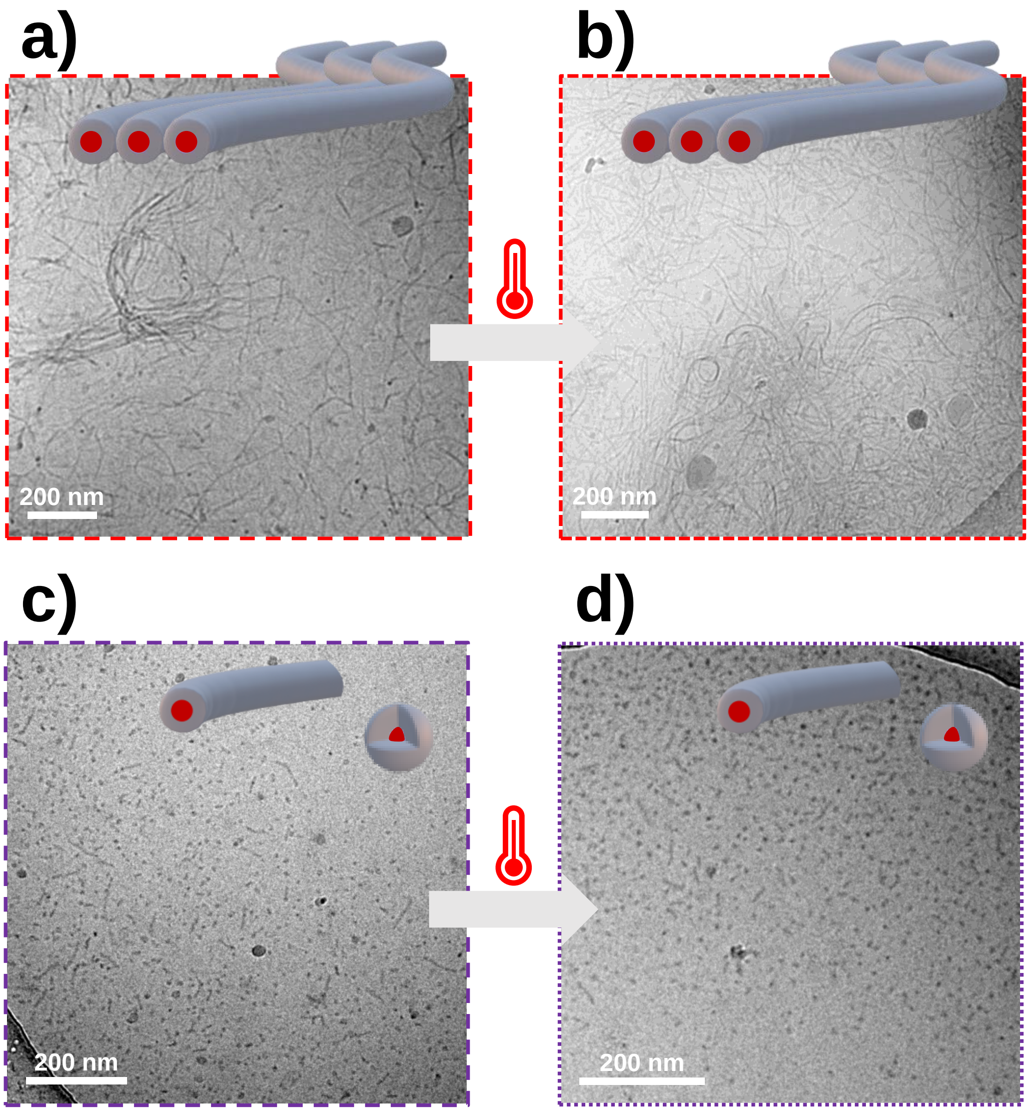

Figure S25: Representative cryo-TEM pictures of the 10 g/L aqueous solutions of (top) **P1** a) not heated and b) heated 10 min at 60 °C and (bottom) the 10 g/L solution **P2** c) not heated and d) heated 10 min at 60 °C. The dark spots are surface contaminations stemming from water crystals.

c) Study of the absorption and emission properties in water.

UV/Vis absorbance and fluorescence measurements at the same molar concentration (7.5 10^{-6} mol/L) were performed by diluting with water the 10 g/L stock solution. For the fluorescence spectra in H_2O, the excitation wavelength selected was 490 nm for PDI-DMAC (TEG-free) polymers and 520 nm for PDI-TEG_2-PDMAc polymers.

These fluorescence experiments at the same molar concentration were also performed with the PDI-PDMAc (TEG-free) polymers **(Figure S26)**. The same trend is observed: when increasing the DP_n, the nanostructures formed are more sphere-like, and a decrease in the I_E/I_M ratio is observed. In this case, despite the presence of spheres, this ratio remains above 1, and this can be due to the non-distorted core of the PDI unit. As the PDI core is planar, the overlap of the orbitals is optimized and leads to a high excimer emission despite the shorter organization range of the nanostructures.[14]

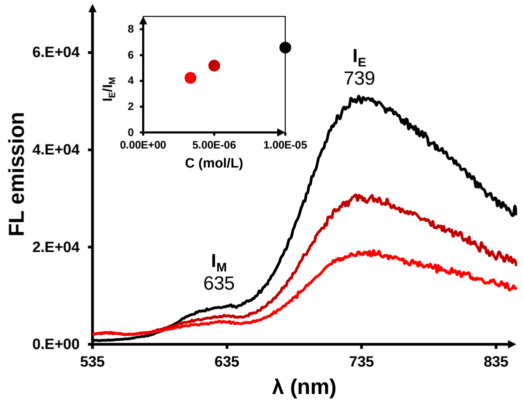

Figure S26: Fluorescence emission spectra n H_2O of **P1-TEG** at 10^{-5} mol/L (black), 5 10^{-6} mol/L (dark red) and 3.3 10^{-6} mol/L (red). The small insert at the top of the spectra shows the ratio between the excimer (E) and monomer (M) emission intensity as function of the concentration. $\lambda_{excitation}$ was set at 520 nm and spectra were recorded at the same conditions.

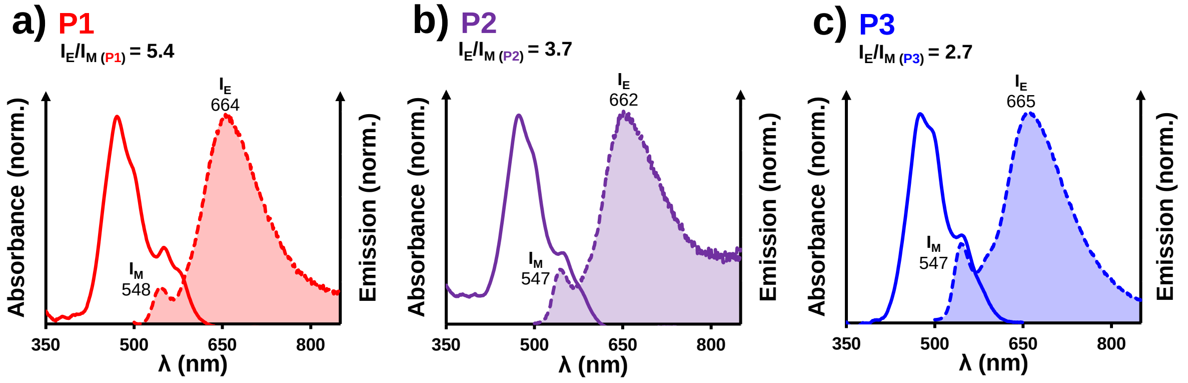

Figure S27: Normalized absorbance (solid line) and emission (dashed lines) spectra at 7.5 10^{-6} mol/L in H_2O of a) **P1**, b) **P2**, c) **P3**. The small insert at the top of the spectra shows the ratio between the excimer (E) and monomer (M) emission intensity. $\lambda_{excitation}$ was set at 490 nm.

5. References

[1] Frisch, M. J.; Trucks, G. W.; Schlegel, H. B.; Scuseria, G. E.; Robb, M. A.; Cheeseman, J. R.; Scalmani, G.; Barone, V.; Petersson, G. A.; Nakatsuji, H.; Li, X.; Caricato, M.; Marenich, A. V.; Bloino, J.; Janesko, B. G.; Gomperts, R.; Mennucci, B.; Hratchian, H. P.; Ortiz, J. V.; Izmaylov, A. F.; Sonnenberg, J. L.; Williams; Ding, F.; Lipparini, F.; Egidi, F.; Goings, J.; Peng, B.; Petrone, A.; Henderson, T.; Ranasinghe, D.; Zakrzewski, V. G.; Gao, J.; Rega, N.; Zheng, G.; Liang, W.; Hada, M.; Ehara, M.; Toyota, K.; Fukuda, R.; Hasegawa, J.; Ishida, M.; Nakajima, T.; Honda, Y.; Kitao, O.; Nakai, H.; Vreven, T.; Throssell, K.; Montgomery Jr., J. A.; Peralta, J. E.; Ogliaro, F.; Bearpark, M. J.; Heyd, J. J.; Brothers, E. N.; Kudin, K. N.; Staroverov, V. N.; Keith, T. A.; Kobayashi, R.; Normand, J.; Raghavachari, K.; Rendell, A. P.; Burant, J. C.; Iyengar, S. S.; Tomasi, J.; Cossi, M.; Millam, J. M.; Klene, M.; Adamo, C.; Cammi, R.; Ochterski, J. W.; Martin, R. L.; Morokuma, K.; Farkas, O.; Foresman, J. B.; Fox, D. J. Gaussian 16 Rev. C.01, **2016**.

[2] A. D. Becke, *J. Chem. Phys.* **1993**, *98*, 5648–5652.

[3] P. J. Stephens, F. J. Devlin, C. F. Chabalowski, M. J. Frisch, *J. Phys. Chem.* **1994**, *98*, 11623–11627.

[4] J. P. Calupitan, T. Nakashima, Y. Hashimoto, T. Kawai, *Chem. – Eur. J.* **2016**, *22*, 10002–10008.

[5] J. P. Dela Cruz Calupitan, O. Galangau, O. Guillermet, R. Coratger, T. Nakashima, G. Rapenne, T. Kawai, *Eur. J. Org. Chem.* **2017**, *2017*, 2451–2461.

[6] N. M. O'boyle, A. L. Tenderholt, K. M. Langner, *J. Comput. Chem.* **2008**, *29*, 839–845.

[7] S. Berruée, J.-M. Guigner, T. Bizien, L. Bouteiller, L. Sosa Vargas, J. Rieger, *Angew. Chem. Int. Ed.* **2025**, *64*, e202413627.

[8] J. Jia, B. Fan, M. Xiao, T. Jia, Y. Jin, Y. Li, F. Huang, Y. Cao, *Macromolecules* **2018**, *51*, 2195–2202.

[9] S. Sengupta, R. K. Dubey, R. W. M. Hoek, S. P. P. van Eeden, D. D. Gunbaş, F. C. Grozema, E. J. R. Sudhölter, W. F. Jager, *J. Org. Chem.* **2014**, *79*, 6655–6662.

[10] Y. Nagao, T. Misono, *Bull. Chem. Soc. Jpn.* **1981**, *54*, 1191–1194.

[11] A. Nowak-Król, F. Würthner, *Org. Chem. Front.* **2019**, *6*, 1272–1318.

[12] A. Mukhopadhyay, K. Liu, V. Paulino, J.-H. Olivier, *Langmuir* **2022**, *38*, 4266–4275.

[13] G. Mellot, J.-M. Guigner, J. Jestin, L. Bouteiller, F. Stoffelbach, J. Rieger, *Macromolecules* **2018**, *51*, 10214–10222.

[14] F. Würthner, C. R. Saha-Möller, B. Fimmel, S. Ogi, P. Leowanawat, D. Schmidt, *Chem. Rev.* **2016**, *116*, 962–1052.
